# From fixed-dried to wet-fixed to live – comparative super-resolution microscopy of liver sinusoidal endothelial cell fenestrations

**DOI:** 10.1515/nanoph-2021-0818

**Published:** 2022-04-20

**Authors:** Karolina Szafranska, Tanja Neuman, Zbigniew Baster, Zenon Rajfur, Oskar Szelest, Christopher Holte, Agata Kubisiak, Edyta Kus, Deanna L. Wolfson, Stefan Chlopicki, Balpreet S. Ahluwalia, Malgorzata Lekka, Marek Szymonski, Peter McCourt, Bartlomiej Zapotoczny

**Affiliations:** Department of Medical Biology, Vascular Biology Research Group, University of Tromsø (UiT), The Arctic University of Norway, Tromsø, Norway; JPK BioAFM Business, Nano Surfaces and Metrology Division, Bruker Nano GmbH, Berlin, Germany; Marian Smoluchowski Institute of Physics, Faculty of Physics, Astronomy and Applied Computer Sciences, Jagiellonian University, Krakow, Poland; ICLab S.z.o.o, Krakow, Poland; Jagiellonian Centre for Experimental Therapeutics (JCET), Jagiellonian University, Krakow, Poland; Department of Physics and Technology, UiT-The Arctic University of Norway, Tromsø, Norway; Chair of Pharmacology, Jagiellonian University Medical College, Krakow, Poland; Institute of Nuclear Physics, Polish Academy of Sciences, Kraków, Poland

**Keywords:** atomic force microscopy (AFM), fenestration, liver sinusoidal endothelial cell (LSEC), scanning electron microscopy (SEM), stimulated emission depletion (STED) microscopy, structured illumination microscopy (SIM)

## Abstract

Fenestrations in liver sinusoidal endothelial cells (LSEC) are transcellular nanopores of 50–350 nm diameter that facilitate bidirectional transport of solutes and macromolecules between the bloodstream and the parenchyma of the liver. Liver diseases, ageing, and various substances such as nicotine or ethanol can negatively influence LSECs fenestrations and lead to defenestration. Over the years, the diameter of fenestrations remained the main challenge for imaging of LSEC *in vitro*. Several microscopy, or rather nanoscopy, approaches have been used to quantify fenestrations in LSEC to assess the effect of drugs and, and toxins in different biological models. All techniques have their limitations, and measurements of the “true” size of fenestrations are hampered because of this. In this study, we approach the comparison of different types of microscopy in a correlative manner. We combine scanning electron microscopy (SEM) with optical nanoscopy methods such as structured illumination microscopy (SIM) or stimulated emission depletion (STED) microscopy. In addition, we combined atomic force microscopy (AFM) with SEM and STED, all to better understand the previously reported differences between the reports of fenestration dimensions. We conclude that sample dehydration alters fenestration diameters. Finally, we propose the combination of AFM with conventional microscopy that allows for easy super-resolution observation of the cell dynamics with additional chemical information that can be traced back for the whole experiment. Overall, by pairing the various types of imaging techniques that provide topological 2D/3D/label-free/chemical information we get a deeper insight into both limitations and strengths of each type microscopy when applied to fenestration analysis.

## 1 Introduction

The unique morphology of liver sinusoidal endothelial cells (LSEC) makes them an excellent platform for testing drug responses [1], tracking the dynamics of the cytoskeleton [2] or even testing the resolution of novel microscopy techniques [3]. The LSEC fenestrations (also called fenestrae) are the main reason for an increasing interest in the research on LSEC morphology. Fenestrations are transcellular pores of 50–350 nm in diameter, typically clustered in groups of a few to a few hundred to form “sieve plates”. Fenestration size is primarily below the resolution of standard optical microscopy, resulting in these pores not being visible in the microscope images. In addition, the thickness of the cell in the sieve plate regions is less than 200 nm, making them ideal test subjects to assess developing super-resolution techniques and data analysis algorithms [3], [4], [5]. However, fenestrations are interesting scientifically not only because of their structure but primarily because of their function. They facilitate extremely efficient passive bidirectional transport of solutes and macromolecules between blood and hepatocytes [6, 7]. The changes in fenestration size and/or number influence the efficiency of this bidirectional transport. The well-fenestrated morphology of LSEC is a marker for proper liver function. Decreased porosity, associated with ageing or liver diseases [8], affects not only the functioning of the liver but also general metabolism [9, 10]. Interestingly, fenestrations rapidly respond to different chemical agents [1, 11] opening a new field on re-opening fenestrations in defenestrated LSEC [2, 12]. Recently, several therapeutic approaches were reported to positively influence the number of fenestrations in young and old animals [12]. The detailed monitoring of fenestrated morphology under different experimental conditions has become an essential component of the research on LSEC. We have recently published a summary of all drugs and agents reported so far to affect fenestration size and/or number in [13].

To achieve proper quantification of the LSEC phenotype, several microscopy techniques have been recently introduced. The size of fenestrations is mainly below the diffraction limit of regular optical microscopy, so their imaging requires the use of super-resolution or nonoptical techniques. To date, scanning electron microscopy (SEM) [9, 14], [15], [16], transmission electron microscopy (TEM) [17, 18], atomic force microscopy (AFM) [9, 19], [20], [21], [22], structured illumination microscopy (SIM) [23, 24], direct stochastic optical reconstruction microscopy (dSTORM) [1, 5, 23, 24], and stimulated emission depletion fluorescence microscopy (STED) [25, 26], were all successfully used to analyze fenestrations in fixed LSEC. Recent advances in AFM [2, 11] and STED [26] have opened new horizons for investigating fenestrations in live LSEC. However, the reported phototoxicity and sample bleaching connected with STED limits the imaging time; thus, AFM currently remains the only technique allowing for prolonged (>1 h) investigation of changes in porosity of LSEC. The abovementioned microscopy modalities require specific sample preparation. For example, high-resolution SEM requires sample dehydration and coating. Data coming from optical nanoscopy are based on formaldehyde (FA)-fixed samples, while AFM on glutaraldehyde (GA)-fixed samples. The influence of sample preparation on fenestrations in LSEC has not been discussed so far. Up to 2012, it was highlighted that fenestrations in wet-(GA)fixed LSEC are larger than in fixed-dried (dehydrated) LSEC [27]. However, the observed net-values of mean fenestration diameter varied largely from the values obtained using novel microscopy, indicating that an update in the comparison is needed.

Correlative imaging can be crucial for avoiding false or misleading conclusions. Several reports have indicated the advantages of coupling various super-resolution fluorescence microscope modalities in one device to provide information about the sample biochemical composition, with a detailed 3D topography provided by AFM [28], [29], [30], [31], [32], [33]. In particular, AFM provided complementary information about the kinetics of amyloid aggregation, while data collected using only fluorescence microscopy (STED) fails to visualize all the products derived from the *in vitro* aggregation of misfolded proteins [32]. It highlights how labeling is a limiting factor in quantitative fluorescence imaging and how the results should ideally be supported with other techniques. Complementary imaging of LSEC was reported in the past, showing the differences in the porosity between the microscopy techniques in fixed-dried (chemically fixed and dehydrated) LSEC [15, 34, 35]. Recently developed photonic chips for correlative light and electron microscopy provide new possibilities for high-resolution imaging of LSEC [36]. So far, comparative imaging of fenestrations has been limited to fixed-dried samples [15, 37]. In particular, the mean fenestration size reported using different techniques varies more than 30% depending on the selected sample preparation method (Suppl. Table in [2]). Such differences may lead to incorrect conclusions about the filtration efficiency of particulate material through fenestrations. For example, large chylomicrons (>∼350 nm) cannot pass the barrier of fenestrations, but chylomicron remnants, LDL or HDL particles can freely migrate through [38]. The reported increase or decrease in the diameter of fenestrations for newly discovered drugs determines their potential in treating liver diseases. It is important to find a way to understand the origins of those discrepancies, allowing for better comparison between reports. The variations might originate from differences between animals (species, age), different sample preparation protocols, imaging techniques, and analysis methods. Recently, we presented a report in which we compared different ways of image analysis allowing for high throughput quantification of fenestrations size and number [39]. The differences between the novel optical microscopic methods for LSEC imaging have been discussed by Øie et al. [24] but the direct comparison including electron microscopy and AFM has not been reported so far. Despite the advantages, all techniques suffer from certain limitations [40, 41]. Optical nanoscopy requires the use of fluorescent labeling. The labeling density, distribution and bleaching all limit the resolution. It should be emphasized that fenestrations are “negative” structures in the context of fluorescence imaging. It means that we are looking at a lack of signal within the pores. Imaging a negative structure requires high density labeling of nearby positive objects (mainly by membrane dyes), and the efficiencies of the binding to those targets influences imaging. AFM is a label-free technique but requires tip-sample interaction, which (when cantilevers with sharpened, i.e., higher resolution tips are used) might alter the investigated structures [19, 42]. Both methods allow for measurements of wet-fixed LSEC or even live LSEC, while SEM needs sample dehydration.

Recent reports have provided great improvement in the imaging of fenestrations in LSEC. However, the direct comparison between them is lacking. Here, we present data from four comparative techniques, where the same cell was imaged using two selected techniques – SIM/SEM, STED/SEM, AFM/SEM, AFM/STED, and AFM/conventional confocal fluorescence. This article aims to discuss the differences between the fenestration diameters obtained by each method. Therefore, we introduced a novel method of correlative imaging in which the same individual fenestrations were measured using both techniques in a one-to-one manner. For accurate measurements we implemented recently introduced methods of image analysis [39]. Moreover, for the first time, we analyze the differences between wet-fixed and fixed-dried samples by comparing SIM/SEM, STED/SEM, and AFM/SEM techniques. We then focus on wet-fixed samples showing the first AFM and STED correlative images of LSEC and discussing the effect of the selected fixation method and permeabilization on LSEC morphology. Finally, we show the possibilities of combining non-super-resolution fluorescent microscopy with AFM for achieving live imaging with additional chemical information.

## 2 Materials and methods

### 2.1 Cell isolation

LSEC were isolated as previously described [20]. Briefly, livers were initially perfused to remove the blood and then digested using Liberase™ (*Roche*). After digestion, the cells were released from Glisson’s capsule into a cold (4 °C) perfusion buffer containing 1% BSA. The obtained suspension of cells was subjected to several centrifugations (including 25–50% Percoll gradient separation for AFM/STED experiments) to separate hepatocytes, remaining blood cells, and nonparenchymal cells. Thereafter, LSEC and Kupffer cells were separated by immuno-magnetic separation using endothelium specific CD146 MicroBeads (*MACS*, *Miltenyi Biotec*, Germany). After isolation, cells were seeded on uncoated glass coverslips and incubated in 5% CO_2_ at 37 °C in EGM-2 cell culture medium (*Lonza*) for 12–15 h for AFM/STED or on fibronectin-coated glass coverslips in RPMI-1640 (Sigma-Aldrich) for 4–6 h for SIM/SEM, STED/SEM, and AFM/SEM. Seeding conditions were optimized according to the specific microscopy requirements, using established methodology [39].

### 2.2 Sample handling

Large gaps, i.e., micron-sized holes in a membrane, were observed in freshly isolated LSEC after seeding. By comparing live and fixed LSEC, we noticed that no new gap formation occurred when LSEC were fixed with FA or GA. By monitoring LSEC morphology live, instead of fixed, when using AFM/conventional fluorescence microscopy, we showed that wet-fixation of cultured LSEC does not damage sieve plates. However, by investigating individual LSEC using AFM on each step of sample preparation, we noticed that thorough rinsing (pipetting) of samples with live or fixed cells could damage the delicate structure of fenestrations within sieve plates. We used warm (37 °C) buffers and fixative agents combined with slow aspiration and delicate rinsing to reduce gap formation.

### 2.3 Fixation

**Wet-fixed LSEC**. FA-fixed LSEC or GA-fixed LSEC terms are used in order to describe chemically fixed cells using aldehydes. In particular, after cell culture medium was removed and 3.6% FA added to LSEC for 15 min (FA-fixed LSEC) or 1% GA for 2 min (GA-fixed LSEC). Then the fixative was removed and cells were kept in phosphate buffered saline (PBS).

**Fixed-dried LSEC**. For 2 h, the sample was post-fixed in McDowell’s solution (4% FA, 1% GA, pH 7.3) [44]. Samples were incubated for 1 h in freshly made 1% tannic acid in PHEM buffer, 1 h of 1% OsO_4_ in H_2_O, dehydrated in ethanol gradient (30, 60, 90% for 5 min each, then 4 times for 5 min in 100% ethanol), followed by two sets of incubation in hexamethyldisilane (HMDS) for 10 min. Finally, samples were left overnight to evaporate.

### 2.4 Staining of actin and cell membrane

3.6% FA-fixed cells were stained for 30 min in PBS using: phalloidin-Atto488 (*Sigma Aldrich*) 1:300, Invitrogen CellMask Deep Red Plasma membrane Stain (*Thermo Fisher Scientific*) 1:500, Invitrogen CellMask Green Plasma Membrane Stain (*Thermo Fisher Scientific*) 1:500; for 5 min using Hoechst 33258 (*Invitrogen*) 1 μg/ml. To avoid any additional effects of the dye on living cell, LSECs were stained after fixation. Cells were not permeabilized using detergent before staining.

### 2.5 Atomic force microscopy (AFM)

Live LSEC measurements were conducted in 25 mM HEPES buffered EGM-2 medium at 37 °C. Fixed cells (3.6% FA for 15 min or 1% GA for 2 min) were measured in PBS with Ca^2+^ and Mg^2+^ at 25 °C. Imaging was carried out in a BioCell™ (*Bruker Nano GmbH, Berlin, Germany*) under ambient atmosphere using Nanowizard 3 AFM system (*JPK Instruments*) and Nanowizard 4 (*Bruker Nano GmbH, Berlin, Germany*) for comparative AFM/STED and AFM/SEM measurements. All images were acquired using the force–distance curve-based imaging mode, a so called “Quantitative Imaging” (QI) (*Bruker Nano GmbH, Berlin, Germany*) according to [11]. Briefly, an independent, 300–800 nm force–distance curve was collected in each image pixel. It was further translated into the topography image, reconstructed for selected loading force, up to the maximal loading force used in the experiment [11]. We performed measurements using different types of commercially available cantilevers: (a) with tips of a radius of 25 nm on cantilevers with a spring constant of 0.1 N/m (SCM-PIC-V2, *Bruker Nano GmbH, Berlin, Germany*) (Figure 3, Supplementary Figures 4 and 5), (b) with tips of a radius of 20 nm on cantilevers with a spring constant of 0.7 N/m (ScanAsyst-Fluid, *Bruker Nano GmbH, Berlin, Germany*) (Figure 6), (c) with tips of a radius of 2–12 nm on cantilevers with a spring constant of 0.03 N/m (MSNL-10 D, *Bruker Nano GmbH, Berlin, Germany*) for AFM/SEM experiments (Figure 4, Supplementary Figure 3), and (d) with tips of a radius of 10 nm on cantilevers with a spring constant of 0.1 N/m (qp-BioAC) (Figure 5). The load force varied from 100 to 350 pN and was adjusted to obtain a clear image of fenestrations at 90% of the load force. The elasticity parameter (apparent Young’s modulus) of LSEC (Figure 3A) was determined using gold-coated V-shaped colloidal probes (polystyrene, diameter 7.9 µm) with spring constants of 0.01 N/m (*Novascan*), as presented in [43]. 8 × 8 µm^2^ areas over the cell nucleus were selected, and 8 × 8 matrices (total 64) of force-distance curves were acquired (Load force of 0.5 nN, load frequency 4 s). The value of the cell elasticity parameter was determined by fitting the Hertz model to each force-indentation curve. The obtained images of the topography, stiffness and elasticity were prepared using JPK Data Processing Software for further analysis.

### 2.6 Structured illumination microscopy (SIM)

Samples were prepared as previously described [19]. Briefly, cells were seeded on fibronectin-coated #1.5 glass-bottom MatTek dishes (*MatTek Corporation*, MA) and fixed for 15 min with 4% FA in PHEM buffer (60 mM PIPES, 25 mM HEPES, 10 mM EGTA, 4 mM MgSO_4_·7H_2_O, pH 6.9). Samples were stored in PBS containing 0.1% FA at 4 °C. Before imaging, cells were stained using 10 μg/ml of CellMask Green (*ThermoFisher*) in PBS for 30 min. Images were obtained at room temperature using a commercial SIM microscope (OMX Blaze system, *GE Healthcare*) with a 60 × 1.42 NA oil-immersion objective (*Olympus*), using oil with a refractive index of 1.514 (*Cargille*) and a 488 nm laser. 3D-SIM image stacks of 1.5–2.0 μm were acquired with a *z*-distance of 125 nm and 15 raw images per plane (five phases, three angles). Raw datasets were computationally reconstructed using SoftWoRx software (*GE Healthcare*), and maximum intensity *z*-projections in *tiff* format were prepared for further analysis.

### 2.7 Stimulated emission depletion microscopy (STED)

Fixed samples were prepared as for SIM. STED imaging was performed at room temperature using a STEDYCON (Abberior compact line) mounted on an inverted optical microscope (Axio Observer, *Zeiss*) equipped with a 100× oil immersion objective with oil with refractive index 1.518. Images were acquired with the STEDYCON smart control software using the 640 nm excitation laser together with the 775 nm STED/depletion laser and 650–700 nm detection. The following parameters were set during STED measurements, dependent on the sample: excitation power: 1–6%, depletion laser power on sample: 210 mW, pixel dwell time: 10–50 µs with double signal accumulation, depletion pulse delay: 0 ns, depletion saturation power: 0.5–3.1%, gate delay: 1 ns, gate width: 6 ns, pinhole: 64 µm. The pixel size was 30 nm for AFM/STED and 39 nm for STED/SEM images. Furthermore, the STED setup was equipped with a Nanowizard 4 system for the complementary AFM investigation.

### 2.8 Scanning electron microscopy (SEM)

After STED, SIM or AFM measurements, the sample was prepared for SEM measurements according to the protocol described in *Methods*, point 2.3. Before imaging, samples were mounted on metal stubs using carbon tape and silver glue to reduce charging and sputter-coated with 10-nm gold/palladium. A commercial SEM system (Sigma HV0307, *Zeiss*) was used to image samples using a 2 kV electron beam. The image size was adjusted to the area measured with SIM, STED or AFM, with a 9–10 nm pixel size. For correlation purposes, slight adjustments (rotate and perspective tools) were performed with graphics software (*Gimp*, version 2.10.24) to obtain perfect matching based on landmarks.

### 2.9 Conventional confocal microscopy

Measurements on fixed LSECs were done using a ZEISS LSM 710 laser scanning confocal unit connected to an Axio Observer Z1 microscope with a 100×, 1.3 NA oil immersion objective. Images were acquired with ZEN 2012 SP1 Black Edition (version 8.1.0.484) software using the 488 and 633 nm excitation lasers. Comparative AFM/LSCM measurements were performed on two independent systems. Marked glass coverslips were used to localize the same position on a sample, which allowed measurements of the same cell with both techniques. AFM analysis was performed prior to confocal fluorescence microscopy measurements. The images were analyzed using Zeiss ZEN (blue edition). The scale and position of the AFM and confocal fluorescence microscopy images were adjusted manually using graphics software (*Gimp*, version 2.10.24).

### 2.10 Data analysis and statistics

For STED/SEM, SIM/SEM comparison, images were processed in Fiji/ImageJ software [44] and segmented using a semi-automated threshold-based method described in detail elsewhere [39]. Briefly, in each image the contrast was individually adjusted, then the image was converted into a binary mask. In particular, for optical nanoscopy the cut-off values were selected carefully, because of the uncertainty connected with the point spread function (PSF) to reflect a point between the maximum intensity and full width at half maximum. After this every fenestration was measured. Parameters such as single fenestration area, fenestration diameter and roundness (ratio of min to max diameter assuming elliptical shape) were measured for each fenestra. This method was shown to be easily implemented and highly reproducible between the users in the assessments of fenestration diameters [39]. For STED/AFM and AFM/SEM an automated machine-learning method was selected, as described in the same report [39]. The advantage of this method is a precise identification of fenestrations at low-resolution (low number of pixels, big pixel size) and high-magnification images [39], here represented by AFM. After the binary mask was prepared, the fenestration measurements were performed using for semi-automated method. OriginPro software (OriginPro 2021, OriginLab Corp., Northampton, MA) was used for data analysis and graphical presentation.

It is important to note that the net values of fenestration size distributions differ between the methods. For example, the mean fenestrations size of SEM differs in SIM/SEM, STED/SEM, or AFM/SEM analyses. This is expected as the selection of analyzed sieve plates was performed in order to perform correlative imaging. To present the mean value of fenestrations in a biological group, several cells for each bio replicate are analyzed by measuring and counting all fenestrations from each cell. Here, we selected individual sieve plates, measured in the optimal conditions of each method. For example, in STED, it is difficult to obtain a high signal to noise ratio in the images of the whole cells and the thermal drift shifts the focus when imaging bigger areas. Therefore, individual sieve plates were measured and compared with SEM. Similarly to AFM measurements, the large view image was collected in the point to point resolution of ∼80 nm to minimize thermal drift. However, 25–40 min per frame is needed. Individual sieve plates were then imaged in high resolution at 15–25 nm per pixel (with up to 15 min per image). Here, the main limiting factor was low throughput, but all techniques allow for comparing the same fenestrations in a one-to-one manner, and the differences between the selected microscopy techniques can be discussed.

## 3 Results

### 3.1 Wet-fixed versus fixed dried I. Comparative SIM/SEM imaging

**Figure 1:**
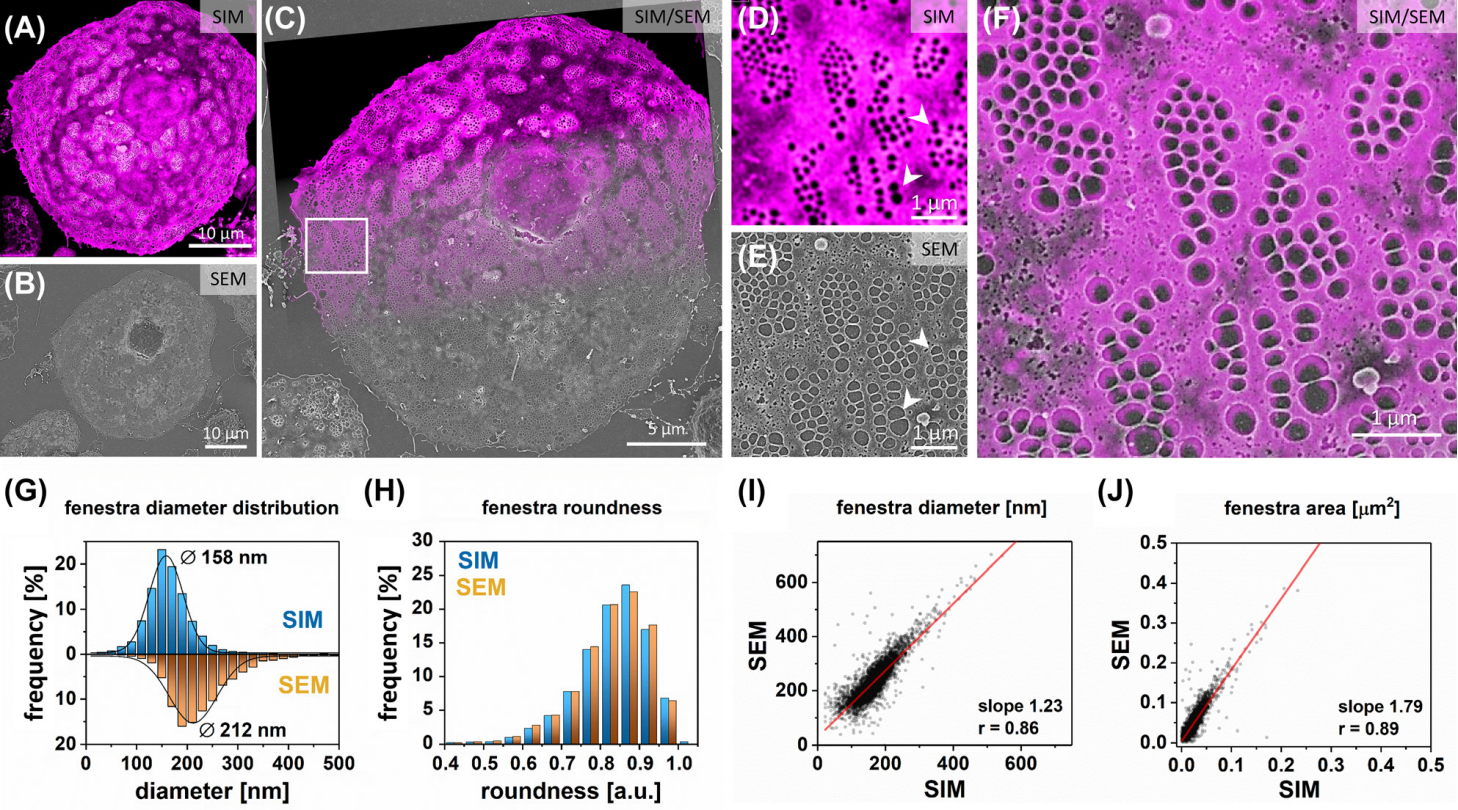
Correlative SIM and SEM imaging. Firstly, SIM imaging was performed on wet-fixed LSEC labeled with CellMask Green; (A) A representative LSEC is presented (Magenta Hot Lookup was used to enhance the contrast of fenestrations in the image). (B) The sample was dehydrated, and the corresponding area was located (using landmarks) and imaged using SEM. (C) Both SIM and SEM images were then correlated. (D–F) High-magnification correlative imaging allows for precise identification of fenestrations within sieve plates using both techniques. Some fenestrations appear merged in SIM but not in SEM (white arrowheads). Fenestrations smaller than 100 nm can be distinguished in SIM (white arrows). 4942 fenestrations were detected and measured in a one-to-one manner using a semi-automated method. (G) Histogram of fenestration diameter distribution. The lines represent fitted Gaussian curves from which the mean values were calculated at the peak of the distribution (158 ± 40 nm and 212 ± 55 nm for SIM and SEM, respectively (*n* = 4942 fenestrations)). (H) Distribution of fenestration roundness measured by SIM (0.83 ± 0.09) and SEM (0.82 ± 0.09) (roundness = ratio of min to max diameter, assuming elliptical shape). A comparison of individual fenestration diameter *I* and area *J* indicate linear and even dilation of fenestrations in fixed-dried LSEC (linear regression slope of *I* = 1.23 (*r* = 0.83) and *J* = 1.79 (*r* = 0.89)). *r* – Pearson’s coefficient.

SIM is a highly versatile optical microscopy method with double the resolution of diffraction limited microscopy. However, it is prone to image artefacts. To investigate this issue, we performed SEM on the same samples that were first examined by SIM. 488 nm excitation was selected to enable the highest optical resolution. In particular, LSEC were labeled with CellMask Green, allowing for detailed imaging of the cell membrane. A representative image of a single LSEC imaged using both SIM and SEM is presented in Figure 1.

The resolution of SIM images allowed for detailed identification of fenestrations in LSEC (Figure 1A). We selected 10–15 representative cells per sample and performed SIM imaging. Using such landmarks as cell shape and arrangement, the same cells were subsequently localized using SEM (Figure 1B). The correlation was performed using scales (calibration using representative scale bars) representative for both microscopes (Figure 1C and F). Using SEM, we identified several individual and small fenestrations located close to one another that appear as one fenestration in SIM (Figure 1D and E). Moreover, some fenestrations near the cell edge are distorted or merged in SEM but not in SIM. However, most of the fenestrations within the sieve plates were identified using both techniques, and the number of artefacts was not significant. The most prominent differences between the two techniques relate to the perinuclear regions of LSEC. In those areas some whole sieve plates were not visible or were dimly visible in SIM. The thick areas of the surrounding cell membrane hampered the proper identification of fenestrations in those sieve plates (Supplementary Figure 1). Therefore, the number of fenestrations per cell calculated with SEM will be larger than with SIM. The next prominent difference is connected with the diameter of the fenestrations. Fenestrations appear significantly larger in fixed-dried samples measured with SEM than in the wet-fixed samples measured with SIM (Figure 1D–F). To quantify this observation, we performed detailed measurements of corresponding fenestrations visualized with both techniques (Figure 1G–J). The analysis of almost 5000 individual fenestrations revealed that the mean fenestration size was 158 ± 40 nm using SIM compared to 212 ± 55 nm obtained from SEM. This corresponds to a 34% increase in diameter between SIM and SEM. There were no observed changes in fenestrations shape, which is reflected in the similar distribution of the roundness parameter – mean value for SEM: 0.82 ± 0.09, SIM = 0.83 ± 0.09 (Figure 1H). The linear relationship between the values of single fenestration area and diameter indicates that the fenestration diameter was evenly dilated for the analyzed group of pores, independently of their size (Figure 1I and J). The resolution of SIM is far lower than SEM, which, together with the possible artifacts from the SIM image reconstruction may affect the measurement of fenestration size. In fact, image reconstruction and processing allowed us for the identification of fenestrations down to 80 nm in diameter (Figure 1G), much smaller than the theoretical resolution of SIM (∼110 nm). Still, those small fenestrations identified in SIM correspond to the fenestrations in SEM (Figure 1 white arrows). In order to achieve even higher spatial resolution, we performed similar experiments using STED – another optical nanoscopy technique which can achieve higher resolution than SIM.

### 3.2 Wet-fixed versus fixed dried II. Comparative STED/SEM imaging

**Figure 2:**
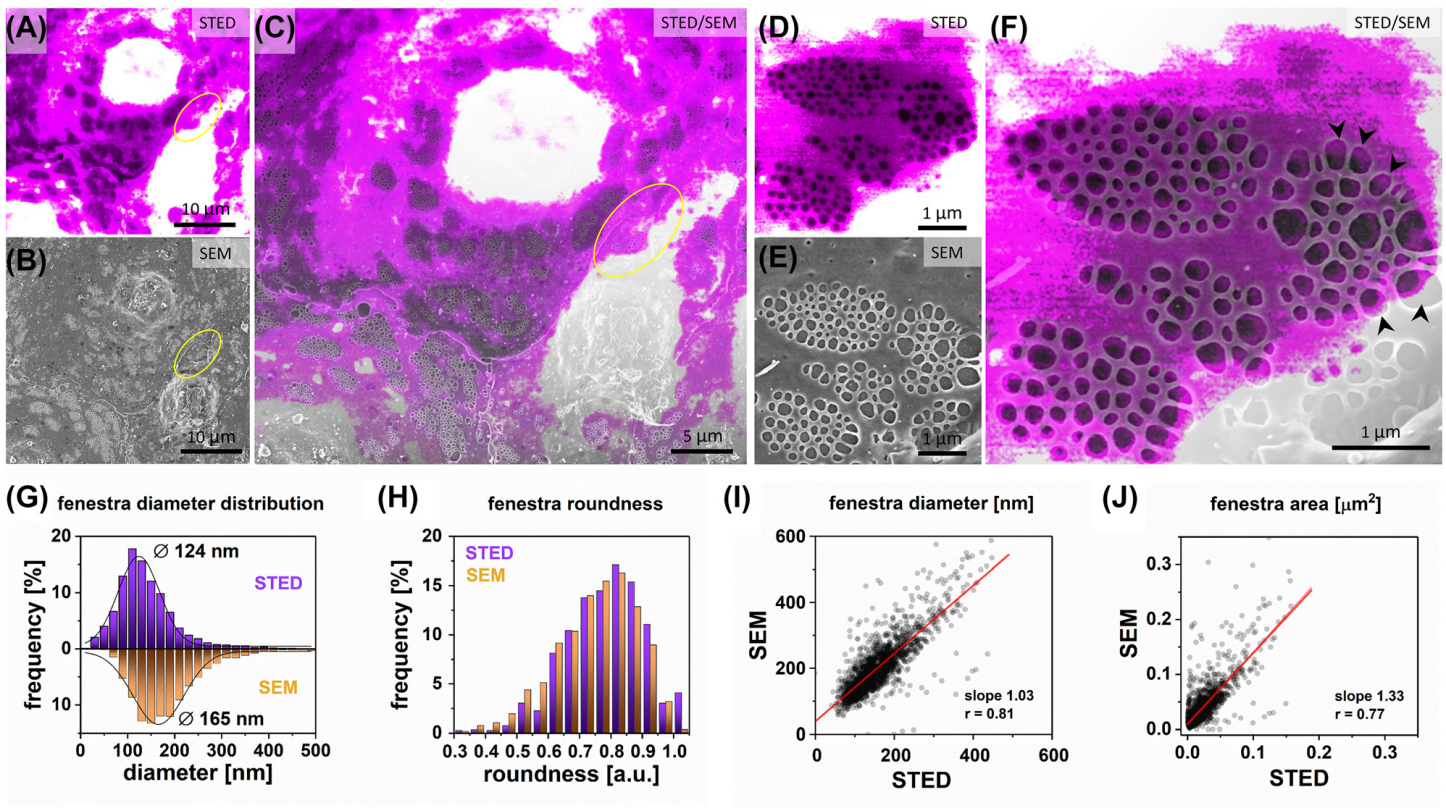
Correlative STED and SEM microscopy. Firstly, STED measurements were performed for wet-fixed LSEC labeled with CellMask Deep Red; (A) A representative STED image of LSEC is presented. To achieve the highest possible contrast for fenestrated regions, areas corresponding to the cell nuclei were saturated (white) due to the high signal intensity. (B) The sample was dehydrated, the corresponding area was localized and measured with SEM. (C) Both STED and SEM images were then correlated. (D–F) High-magnification images allow for precise identification of all of the fenestrations within sieve plates. Fenestrations in the center of the sieve plate were less dilated than those on the edges (arrowheads). 1909 fenestrations were identified in the collected images and analyzed in a one-to-one manner. (G) Histogram of fenestration diameter distribution. The black lines represent fitted Gaussian curves from which the mean diameter was calculated at the peak of the distribution (STED 124 nm ± 44 nm, SEM 165 nm ± 64 nm). (H) Distribution of fenestration roundness measured by STED and SEM. A comparison of individual fenestration diameter *I* and area *J* indicate uniform dilation of fenestrations in SEM.

We used CellMask Deep Red membrane stain to visualize the cell membrane surrounding fenestrations, as suggested in another report [26]. The advantages of STED microscopy cover the lack of reconstruction artifacts and the possibility of selecting small areas with a few sieve plates. It allows for decreased illumination time of LSEC and thus reduces bleaching of the surrounding area. The STED beam allows for precise illumination compared to the larger areas illuminated with the grid pattern in SIM; although the energy of the illumination is usually higher. Despite the growing number of new STED dyes, the selection is still somewhat limited. CellMask Deep Red provides both good dye density and resistance to bleaching allowing for detailed investigation of fenestrations. A representative STED image of LSEC shows the ultrastructure of fenestrations (Figure 2A).

Similarly to previous experiments, we used landmarks to localize the previously measured area on fixed-dried samples using SEM. The correlative image allowed identification of corresponding fenestrations using both imaging techniques (Figure 2C and F). Similarly to SIM/SEM, some artefacts were observed. We identified rupture and merging of some fenestrations (Supplementary Figure 2). Moreover, fenestrations in the perinuclear region or in the higher regions of cells (that are out of the plane of focus) can be omitted in STED analysis (Figure 2A–F). The detailed analysis of 1909 fenestrations detected by both techniques allowed for the direct comparison (Figure 2G–J). The mean fenestration diameter calculated for STED (124 ± 44 nm) and SEM (165 ± 64 nm) corresponds to an overall 33% increase in diameter and is similar to the SIM/SEM experiment. It is important to note that the net values obtained using SIM/SEM and STED/SEM differ. This is expected, as the fully random selection of the cells and the analysis of all cells’ fenestrations was not applied for the analysis, however, all obtained images were used for analysis and no measurements were discarded afterwards; (see *Methods*).

The linear regression slope of 1.03 and *r* of 0.81 reflects the increase in fenestration diameters observed in SEM. Two groups of fenestrations were recognized: the majority of fenestrations were uniformly enlarged due to drying, but also some individual fenestrations enlarged to more than twice their STED-measured size and had an elongated shape. The shift in fenestrations shape in SEM can be observed in both mean roundness values – STED: 0.79 ± 0.11, SEM: 0.75 ± 0.13 and difference in the roundness distribution below 0.7. These deformed fenestrations are located at the edges of sieve plates and in the areas where the cell is either significantly thicker (nuclei region) or thinner (edge of the cell) than the average region with sieve plates (Figure 2F, arrowheads).

### 3.3 Wet-fixed versus fixed dried III. Comparative AFM/SEM imaging

#### 3.3.1 From living to fixed LSEC – AFM study on sample preparation

**Figure 3:**
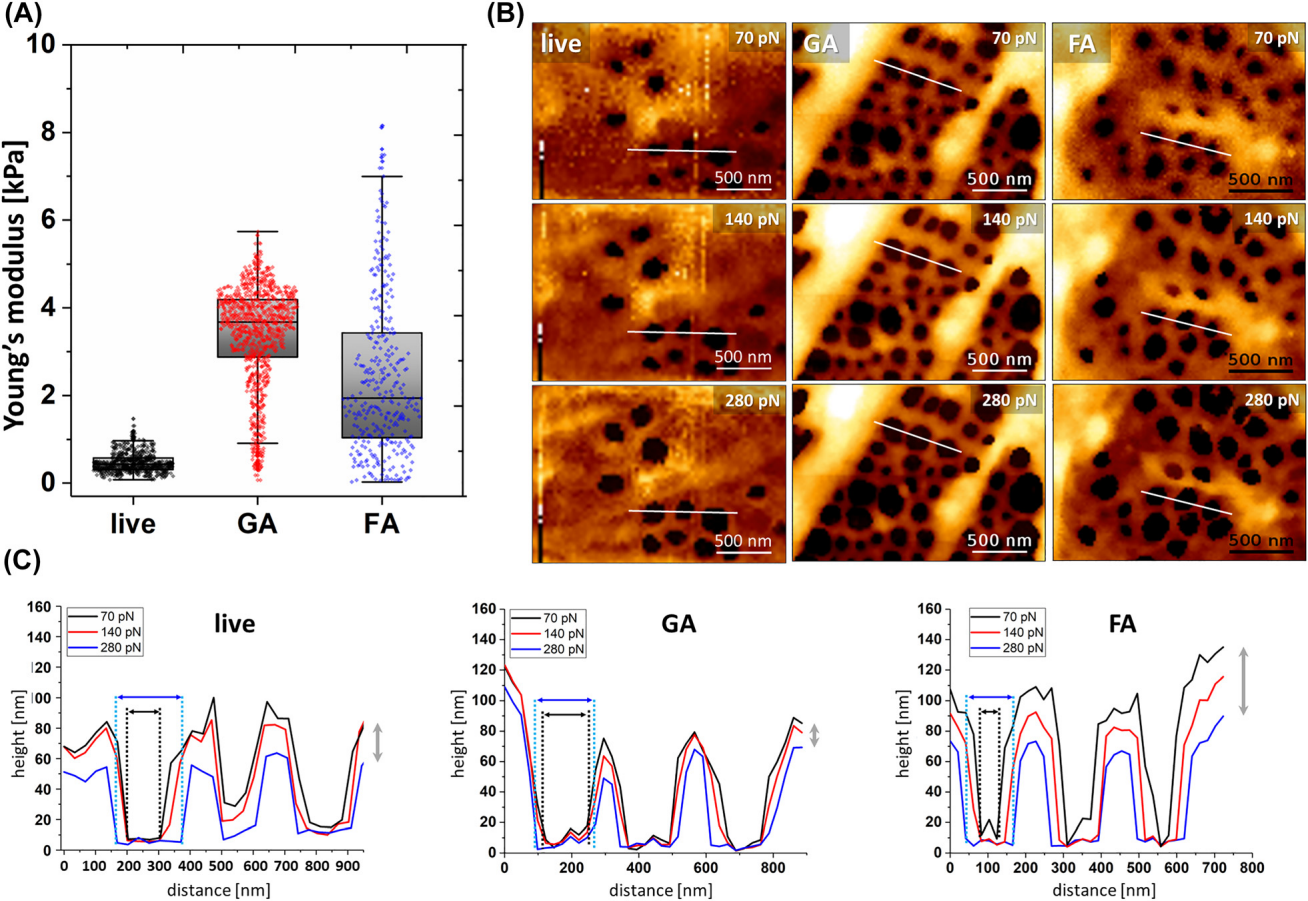
The difference in mechanical properties of LSEC and fenestrations in living, FA-fixed, and GA-fixed LSEC. (A) The apparent Young’s modulus of cells (*n* ≥ 15 for each group, 64 curves per cell) was obtained for a colloidal cantilever for a load force of 200 pN. (B) Images of selected sieve plates measured using QI AFM reconstructed for a load force of 70 pN, 140 pN, and 280 pN. (C) The tip-induced enlargement of fenestrations presented as a cross-section of selected fenestrations collected for the same area of the images reconstructed for different load forces. Grey arrows indicate the tip-induced squeezing of the membrane surrounding fenestrations in the *z*-axis. Black and blue arrows indicate the boundaries of fenestration obtained for 70 and 280 pN, respectively.

In optical microscopy, the optimal fixation methods are limited by the autofluorescence of the agents and interactions with the fluorophores. Therefore, in most cases formaldehyde is the preferable fixative agent over for example, glutaraldehyde. Moreover, most antibody-based staining methods require a permeabilization step which can destabilize the cell membrane and affect fenestration measurement. To study those effects and investigate how different steps in sample preparations affect fenestrations we used the advantages of AFM. This label-free technique can be used in a wide range of environmental conditions and AFM images of LSEC have been reported for living, wet-fixed, and even fixed-dried cells. Therefore, before performing correlative imaging with AFM, we tested standard procedures in preparation of LSEC for microscopy, namely wet fixation using FA or GA and Triton X-100 permeabilization. The differences in fixation methods and their influence on the quality of super-resolution fluorescence imaging were discussed in detail by Whelan and Bell [45]. The authors presented optimized protocols for formaldehyde, glutaraldehyde, and methanol fixation, showing that the latter gives unsatisfactory results for actin staining. Because fenestrations are reported to consist of actin [23], we did not fix LSEC using alcohols. According to the Whelan and Bell protocols and our previous observations [20], we selected 1% GA (2 min) and 3.6% FA (15 min) to investigate changes in the morphology of LSEC (Figure 3).

Firstly, we performed force mapping of live LSEC using a colloidal probe. Then, we analyzed the data for low loading force (200 pN), which corresponded to 50–200 nm indentation to uncouple the information about the elasticity of the deeper layer of the sample (e.g., cell nucleus, glass substrate) and to investigate only the cortical layer of the cell. We observed stiffening of the cortical layer for both fixatives compared to living cells (Figure 3A). The mean apparent Young’s modulus of GA-fixed cells increased 7.2 fold, while a mean 5.2 fold increase was observed for FA-fixed LSEC. GA resulted in a narrower distribution of elasticity than FA. In the next step, using cantilevers with sharp tips, following the methodology described in [19], we investigated fenestration diameters of FA- and GA fixed samples. QI AFM enables image reconstruction for any load force: from near the contact point (“zero force”) up to the maximum load force used in an experiment. In particular, to analyze the load force dependence for both fixative agents, we compared 70, 140 and 280 pN load forces applied in each pixel-point of the image (Figure 3B and C). The first point corresponds to near the contact point, where fenestrations can be distinguished. The following two points double the force, where the last point of 280 pN corresponds to 90% of the maximal indentation force used in the experiment and allows for stable imaging. FA-fixed LSEC requires slow-scanning using a minimal load force to avoid fenestration dilation. A >25% increase in the mean fenestration diameter in FA-fixed LSEC was observed with increasing load force. This effect corresponds with the deformation observed in the fenestration profile for both live and FA-fixed cells (Figure 3C). In contrast, GA-fixed LSEC presented much weaker load force dependence resulting in less than 10% change of fenestration diameter when comparing load forces in the contact and maximal indentation points.

#### 3.3.2 Combining AFM with SEM

**Figure 4:**
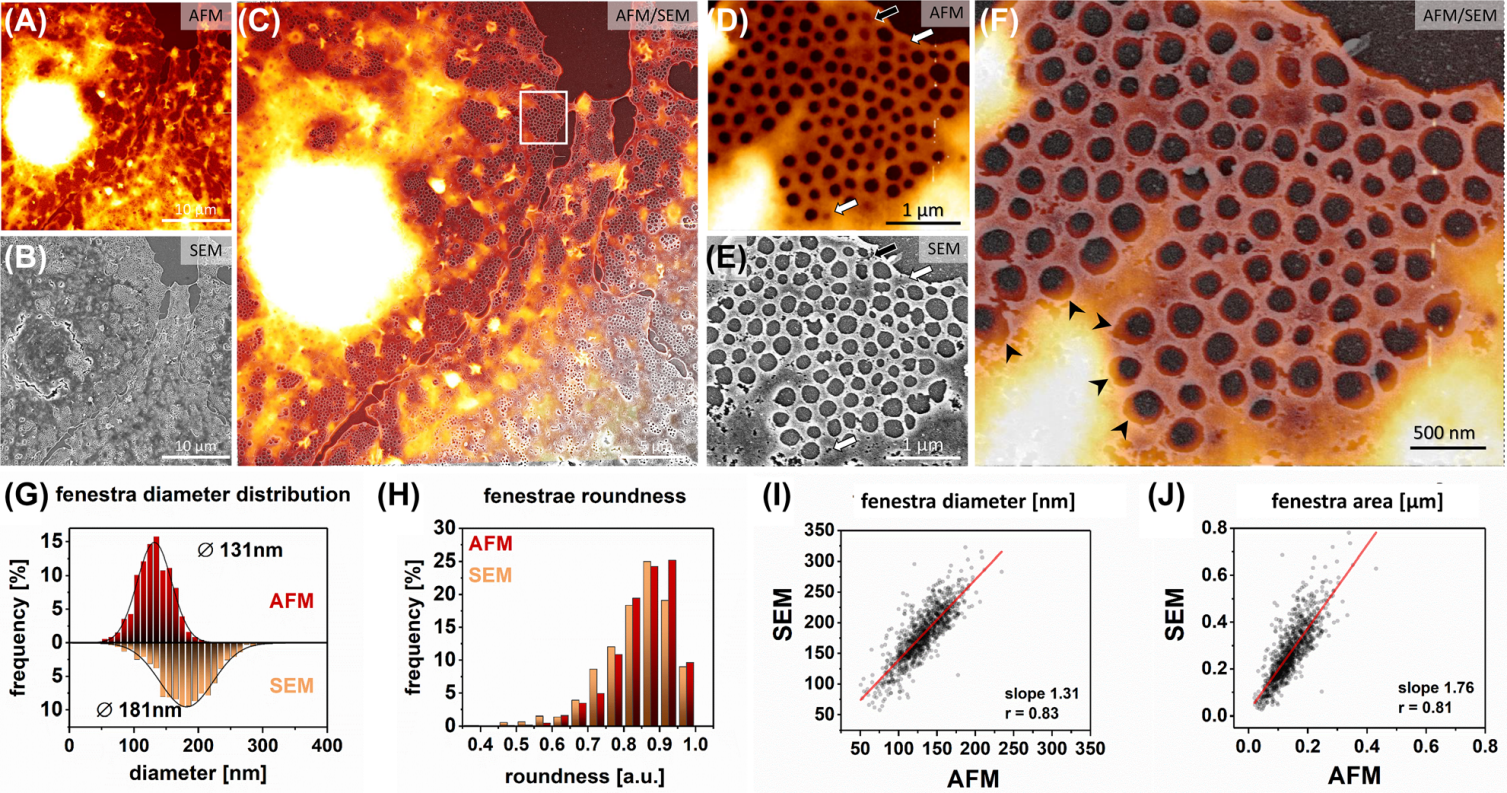
Correlative AFM and SEM microscopy of LSEC treated with cytochalasin B. Firstly, AFM measurements were performed for GA wet-fixed LSEC; (A) A representative AFM image of LSEC is presented. (B) The sample was dehydrated, the corresponding area was localized and measured with SEM. (C) Both AFM and SEM images were then correlated. (D–F) High-magnification image allows for precise identification of all of the fenestrations within a sieve plate. Fenestrations in the center of the sieve plate were less dilated than those on the edges (arrowheads). Some closed fenestrations identified in AFM became open (black arrow) or are not distinguishable (white arrow) in SEM. 1081 fenestrations were identified in both methods and analyzed in a one-to-one manner. (G) Histogram of fenestration diameter distribution. The black lines represent fitted Gaussian curves from which the mean diameters were calculated at the peak of the distribution (AFM 131 nm ± 31 nm, SEM 181 nm ± 48 nm). (H) Distribution of fenestration roundness measured by AFM and SEM. A comparison of individual fenestration diameter *I* and area *J* indicate uniform dilation of fenestrations in SEM.

In the final comparison between the wet-fixed and dehydrated samples, we performed AFM/SEM experiments. Neither requires sample labeling and both provide high resolution. Here we used GA fixation, as it shows stronger and more uniform fixative properties than FA and it is widely used in sample preparation protocols for SEM. We applied measurements using sharpened cantilevers (2–12 nm). It was reported that these provide high-resolution imaging with a steep cut-off on fenestrations boundaries, allowing for precise measurements [19]. We performed AFM/SEM experiments to verify whether the differences in fenestration size in SIM/SEM and STED/SEM experiments are due to the PSF (effectively reducing the measured fenestration diameter in SIM and STED) or dehydration and cell body shrinkage (effectively dilating fenestrations in SEM). The analysis of the AFM/SEM correlation is presented in Figure 4. Cytochalasin B treated LSEC were investigated to increase the number of fenestrations and imaging efficiency. Cytochalasins were reported to increase the number of fenestrations up to three times without any major influence on fenestration diameters [13, 25]. They disrupt the actin cytoskeleton, which lowers overall cell stiffness by removing stress fibers, but does not affect the AFM imaging in used QI (force-dependent) mode of GA-fixed LSEC (Supplementary Figure 5).

The correlation between the methods allowed for precise identification of fenestrations using both techniques. High magnification images of individual sieve plates allowed for one-to-one comparison of fenestrations dimensions. Analysis of 1081 fenestrations confirmed previously the observed enlargement of fenestrations in SEM. The mean fenestration size obtained using AFM was 131 ± 31 nm compared to 181 ± 48 nm for SEM (Figure 4G). This corresponds to a 38% increase in diameter after dehydration. As mentioned above, the net differences in mean size values correspond with the analysis of selected sieve plates (see *Methods*). Similarly to SIM/SEM and STED/SEM, we observed enlargement of the fenestrations at the edges of sieve plates after dehydration (Figure 4F, arrowheads). In addition, we observed that in some sieve plates located in the perinuclear zone – where the cell height is above 400 nm – the shape of fenestrations within is distorted (Supplementary Figure 3). Moreover in those areas, two layers of fenestra-like structures were observed forming structures resembling reported “fenestration labyrinths” [46]. Both techniques resolved even the smallest fenestrations and their number was preserved. The only detected difference can be connected with the open and closed (or fused) state of fenestrations, described recently in [1]. In an AFM image (Figure 4D), some fenestrations can be observed as just invaginations in the cell membrane (arrows), and not as transcellular pores. After dehydration for SEM imaging (Figure 4E), those close fenestrations either became open (black arrow) or remained closed but indistinguishable in the SEM image (white arrows). The newly opened fenestrations in SEM could be identified by the sharp edges and less circular shape. We observed a shift in the roundness distribution towards more circular in AFM (0.92 ± 0.09) compared to SEM (SEM: 0.85 ± 0.09) (Figure 4H). The linear relationship between the values of single fenestration area and diameter remains independent of their size (Figure 4I and J). This observation is similar in all tested methods.

### 3.4 Wet-fixed versus wet-fixed. Comparative AFM/STED imaging

**Figure 5:**
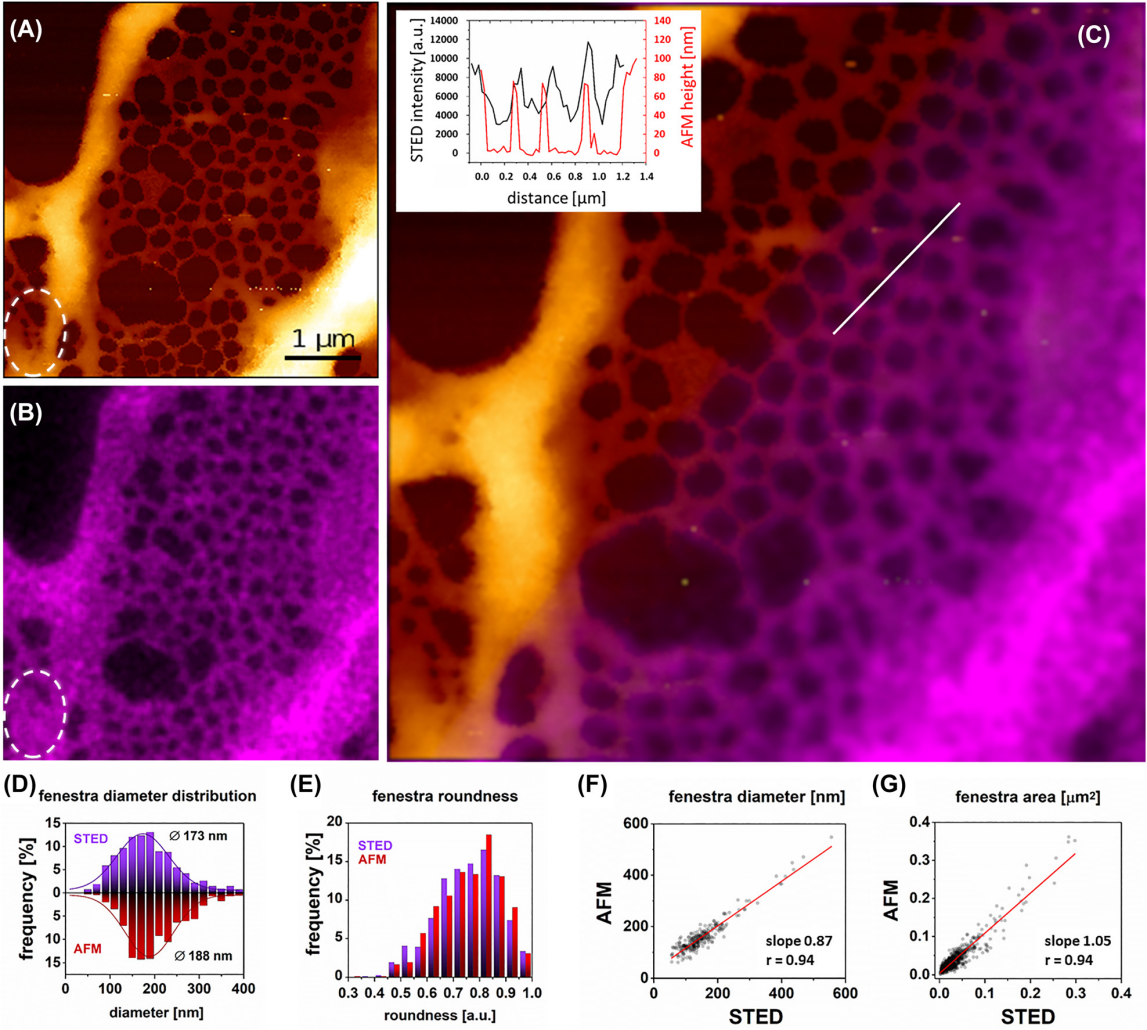
Correlative AFM and STED microscopy. Comparative AFM and STED of FA-fixed LSEC treated with cytochalasin B using a device prototype that allows direct measurements using both techniques in one setup. Firstly, AFM measurements were performed for FA-fixed LSEC (load force 70 pN) labeled with CellMask Deep Red. (A) High-resolution image of a sieve plate in the LSEC periphery measured using AFM. (B) The corresponding area measured using STED. Fenestrations as small as 50 nm can be distinguished with AFM but not with STED (white dashed circle). (C) presents the correlative image of the same area measured using STED and AFM. The inset represents a cross-section of selected fenestrations (white line in (C)). 709 fenestrations were identified in the collected images and analyzed in a one-to-one manner. (D) Histogram of fenestration diameter distribution. The black lines represent fitted Gaussian curves from which the mean diameters were calculated at the peak of the distribution (STED 173 nm ± 58 nm, AFM 188 nm ± 54 nm). (E) Distribution of fenestration roundness measured by STED and SEM. A comparison of individual fenestration diameter *F* and area *G* indicate uniform and precise measurements of fenestrations using both techniques.

AFM and STED comparative measurements of LSEC were performed on a single device. We investigated FA-fixed LSEC, as a standard fixation approach for STED measurements. We selected CellMask Deep Red staining to label the LSEC plasma membrane, which Di Martino et al. [26] previously suggested as suitable for fenestrations and Brunetti et al. established that it worked well with STED [47]. Permeabilization of the cell membrane prior to staining is not required for the selected dye. As for AFM/SEM experiment, we investigated cytochalasin B treated LSEC. Comparisons of AFM and STED of the same area shows a good correlation between obtained images (Figure 5).

Both techniques enabled precise measurements of the number and size of fenestrations. The cross-section in the same location showed a good correlation between both techniques. AFM provides much steeper boundaries between fenestrations, which allow easier analysis with lower error [39]. The fenestrations boundaries are less sharp for STED images due to the blurring related to the PSF. It results in low accuracy in identifying the smallest fenestrations (Figure 5, white circle). Nevertheless, we obtained similar fenestration diameter distributions using both techniques (Figure 5D). Analysis of 709 fenestrations resulted in a mean fenestration diameter of 173 ± 58 nm for STED and 188 ± 54 nm for AFM, which corresponds to a <10% increase due to deformation caused by AFM tip. The shape of fenestrations remains the same for both techniques which is indicated by the similar mean roundness values of 0.75 ± 0.11 and 0.76 ± 0.11 for STED and AFM, respectively. The slope in the single fenestration area comparison indicate that fenestration enlargement is the most prominent for large holes (Figure 5G).

### 3.5 AFM-based measurements combined with conventional fluorescence microscopy – a proposed approach for LSECs fenestrations imaging

**Figure 6:**
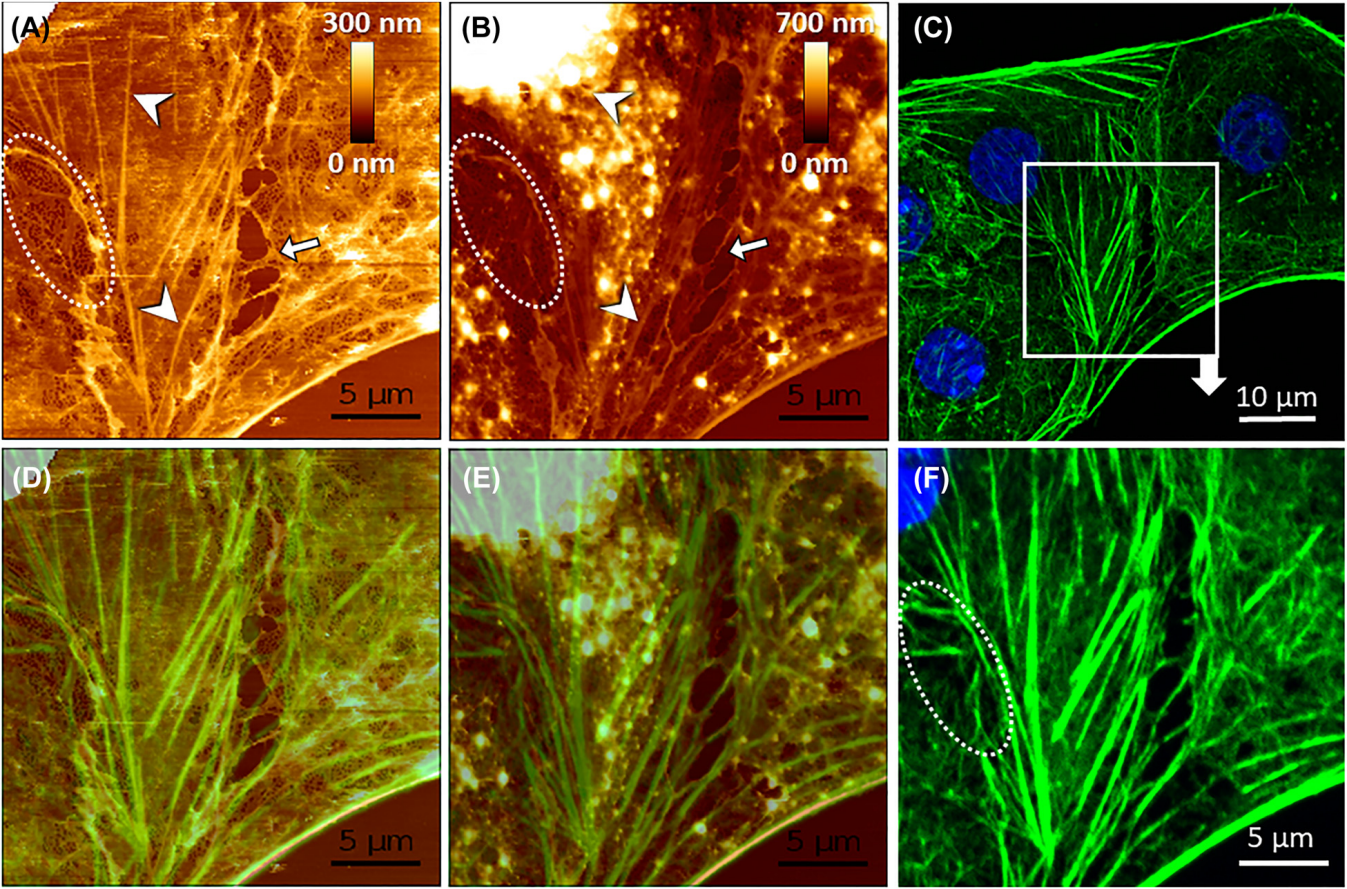
Correlative AFM and conventional fluorescence microscopy of LSEC. (A) The topography of live cells was measured using AFM. (B) The same area measured after fixation with 1% GA. (C) LSEC were then labeled with phalloidin-Atto488 (green) for actin and Hoechst 33258 (blue) for cell nuclei, and the same area was localized using landmarks. (F) The area of interest corresponding to AFM measurements was selected. (D and E) present merged AFM and fluorescence microscopy images made, respectively, for live and fixed LSEC. Comparative data allow identifying actin stress fibers in live cells (white arrowheads), which visualization is hindered in fixed cells. Fenestrations can be identified as dark spots in AFM images of both live (A) and fixed (B) LSEC (dotted-line circle) but not in fluorescence microscopy images (F). Different sizes and position of gaps in live and fixed cells corresponds to cell migration prior to fixation (white arrows).

Here, we present an experiment in which LSEC dynamics was tracked using AFM. The sample was finally fixed for additional non-super-resolution imaging, namely confocal fluorescence microscopy (Figure 6) at the end of the experiment. Such an approach combines super-resolution information about the dynamics of live LSEC with identifying biological structures arising from fluorescence microscopy. Even without super-resolution fluorescence imaging, such an approach can be beneficial for experiments on LSEC. Other optical techniques, such as deconvolution microscopy or spinning disk microscopy can be also used. In particular, we used landmarks such as cell shape and arrangement to identify the areas of interest when transferring samples between AFM and confocal microscope. At first, we performed an experiment in which live LSEC were measured using semi-stiff cantilevers. Such cantilevers allow for fast imaging with a load force of 1–2 nN, enabling visualization of the thick actin cytoskeleton beneath the cell membrane (Figure 6A). Afterwards, the sample was chemically fixed directly on an optical microscope. The high-resolution imaging was repeated in the same area (Figure 6B). Finally, the AFM measurements were followed by staining cells for actin and performing correlative fluorescence imaging of the same regions (Figure 6C and F).

We selected an area of the interconnection of three live cells, where fenestrated morphology was observed. The presented AFM image allowed visualization of sieve plates and fenestrations (Figure 6, white dotted-line circle). Thick stress fibers lining the cell can be easily distinguished as bright lines (Figure 6, arrowheads). After fixation, cells became stiffer and the AFM image corresponds more to the superficial topography of LSEC; fenestrations could still be noticed in the sieve plates, but most of the stress fibers were less prominent and partly covered with overlaying structures. The slight misalignment in AFM images for live and fixed cells is caused by the line-by-line acquisition of AFM in 20 min per frame. During this time, the cell cytoskeleton was continuously rearranging, resulting in, e.g., different sizes of individual sieve plates or changed positions of the gaps between images (Figure 6, white arrows), similar to previous reports [2, 11]. To reduce this effect, we measured and finally fixed LSEC after ∼20 h post-seeding, where the rearrangement of the cellular cytoskeleton was shown to be relatively slow [11]. Finally, we stained cells using phalloidin-Atto488 for actin, and we observed a significant correlation of fluorescence and AFM images. Thick actin fibers remained in the exact positions as observed using AFM for live LSEC.

## 4 Discussion

In this report, we presented a first direct comparison of several super-resolution microscopy measurements of fenestrations in LSEC. Labeling the cell membrane is a well-established approach, allowing the comparison of the labeled cell and the lack of signal within the open, transcellular pore. Fenestrations can change their size in live LSEC *in vitro* by up to 200% [2], but the overall distribution of diameters is preserved after fixation. Still, different values of mean fenestrations sizes have been reported in various reports, with a pattern suggesting a dependence on the selected microscopy technique (SI_Table 1 in [2]). The accurate determination of fenestration size is crucial for understanding the filtration function, as only particles smaller than fenestrations can passively pass from the bloodstream into the space of Disse within the liver [38]. Here, we provided a detailed comparative analysis of fenestration diameters obtained using a combination of different types of microscopy. We want to emphasize that the presented mean fenestration size values are not absolute and do not describe mean fenestration size in the whole population of murine LSEC. The variations were observed because not all fenestrations from each cell were compared. The comparison occurs rather between individual sieve plates that were chosen in the context of efficient co-localization between techniques. Proper investigations using individual techniques can be found elsewhere (AFM [19], SIM [48], STED [25], SEM [49], and TEM [50]). AFM and TEM independently showed similar data on fenestration size distribution. TEM however is not widely used in the evaluation of drug treatment and therefore is not included in this manuscript. TEM could be a perfect tool for the comparison of *in vitro* versus *in vivo* data on fenestrations, including their 3D structure [45]. The differences in the observation of fenestrations between cell culture and tissue are large (SI_Table 1 in [2]), probably because of complex sample preparation [16]. The use of novel TEM methods, including freeze-fraction sample preparation and FIB-SEM could be a part of a future study. We limited our EM studies in this paper to the SEM modality, where we selected individual well-fenestrated cells and compared fenestrations in a one-to-one manner. It allowed us to assess the origins of the discrepancies between the reports obtained using different microscopy modalities. Firstly, we showed that there are differences between fenestration diameter obtained for wet-fixed and fixed-dried LSEC. Our data are in contrast to that presented in [37] where an almost 30% reduction of fenestration diameter was reported for wet-fixed to fixed-dried fenestrations. However, that data was affected by the nascent and still developing AFM technique. The use of contact mode requires a relatively large load force; paired with limited microscope stability, it resulted in image artefacts as streaks and shadowing. The obtained mean fenestration diameter was 269 ± 44 nm using AFM, well above the values recently shown using gentler imaging modes of modern AFM microscopes [19, 42]. The influence of dehydration on fenestration diameters was also discussed in liver tissue [16]. The authors reported up to a 30% decrease in the size of small structures in tissue blocks measured with SEM and advised against the analysis of size using this technique. In tissue samples, dehydration causes collapse and shrinking of the tissue blocks, which results in the observed decrease of fenestration diameter. Our data comes from isolated LSEC measured *in vitro*, and the observed change in fenestration size is in the opposite direction. Cell surface integrins keep cells well-spread on the surface of the glass coverslip. When dehydrated, cells remained in the same shape and size, confirmed by our correlative imaging (Figures 1, 2 and 4). Individual fenestrations are kept in place by the actin cytoskeleton, which constitutes the stiff scaffold of fenestrations. The precise composition of the protein system that binds actin and the cell membrane is currently unknown in LSEC, but previous reports showed the involvement of spectrin [1] and myosin [51]. Together with the membrane composition [52], this structure regulates the fine-tuning of the fenestration size. As a result of cell body dehydration, the dilation was observed, suggesting that the connection between the cell membrane and the cytoskeleton scaffold may be affected by the created extra tension. The level of changes depends on the thickness of the surrounding cell. Thick areas consist of more water and after drying were affected much more than thin and flat areas within sieve plates (Figures 2F and 4F). Also, dehydration may create more tension close to the cell edges which sometimes results in stretched sieve plates with elongated fenestrations. Thereby, an overall 30–40% increase was observed compared to wet-fixed samples and those differences were documented independently using SIM, STED, and AFM. Altogether, small changes in fenestration diameters between the biological groups should be interpreted carefully, as the microscopy techniques combined with analysis methods (pixel size) are burdened with 10–20 nm error [39]. The correlation between fenestration diameter distributions between STED and AFM was within the margin of error. Furthermore, all wet techniques (SIM, STED, AFM) showed similar differences after preparation for SEM imaging. Therefore, in order to compare results between the methods we advise using a coefficient of *0.75 × mean fenestrations size in SEM* to calculate the expected mean fenestrations size for wet techniques, assuming a similar preparation protocol. Note that the elongation of fenestrations at the edges of sieve plates causes the standard deviation to be always wider in SEM.

Similarly, the differences in the porosity between the methods can be expected. Optical techniques would usually show lower values of fenestration frequency (the number of fenestrations per area) or porosity (the ratio of the total area covered with fenestrations to the area of cell/image), as those techniques have difficulties resolving fenestrations in the thick, perinuclear area. In contrast, both AFM and SEM showed to be much more efficient in visualizing fenestrations in these areas, including visualization of previously reported “fenestration labyrinths” [46]. Observation of the fenestration labyrinths in AFM indicates that these structures are not artificially induced after sample dehydration. Their biological role is not described yet, however, they have not been reported in LSECs *in vivo* so far.

For all described techniques, problems in the detection and measurement of fenestrations can be related to the difficulties in the detecting of fenestration edges. In label-free techniques, such as AFM and SEM, the exact edge of the fenestration can be easily defined. On the other hand, in label-dependent techniques, such as STED and SIM, blurring related to the point spread function is observed (Figure 5C, inset). Moreover, the size of fenestrations is close to the achievable resolution of those techniques, which together with the PSF effects, may contribute to the lack of detection of some smaller pores. Small fenestrations (below 100 nm) may also be omitted due to the sampling problem – pixel size in the optical methods is usually scaled to the resolution and may result in undersampling.

Interestingly, we measured fenestrations with a size smaller than the theoretical resolution limit of our SIM system, but these results agree both with earlier SIM data shown by Cogger et al. [53], and, more importantly, with the underlying ‘ground-truth’, higher resolution correlative SEM images. The theoretical resolution of SIM in our setup (excitation/emission 488/525 nm, 1.42NA objective) calculated using the Rayleigh criterion is about 110 nm; however, less strict criteria such as the Abbe limit or Sparrow limit show our theoretical resolution closer to 80 nm, which aligns closer to our actual measurements. Demmerle et al. highlighted the difficulties in assessing the resolution in super-resolution imaging and in comparing between them [54]. In our case, there are several possible explanations for why we observed smaller than expected fenestrations, however, we are not certain of the definite cause here. Reconstruction of raw SIM data into super-resolution images requires a significant amount of post-acquisition image processing, and the final image is presented as a *z* projection, which in combination with not truly isotropic SIM data could make fenestrations appear smaller. The threshold used for image segmentation may also influence the measured diameters, as the exact location of the edge of a fenestration can be interpreted in multiple ways. Finally, it is possible that fenestrations may benefit from appearing as negative objects, with strong signal coming from the positively-stained objects (i.e., membrane) around them, as opposed to trying to image and measure weakly-fluorescent, isolated positive objects; having a better signal-to-noise ratio generally results in better reconstruction and resolution.

Our correlations were independent of cytochalasin B treatment. Other reports, applying STED and SEM individually, showed no changes in fenestration diameters of LSEC treated with cytochalasin B [26, 49]. It indicates that the level of actin polymerization does not additionally alter fenestration diameters during dehydration. Altogether, it allows us to conclude that the dehydration of samples in fixed-dried LSEC is significant, but constant, allowing for comparison between samples prepared, measured, and analyzed in the same way. We emphasize the need to analyze a large number of fenestrations (to avoid bias due to the large cell-to-cell differences). Recently developed automatic image analyses allow for the measurement of thousands of fenestrations a relatively quickly [39] or precise measurements of fenestrations in low-resolution images [55].

A comparison of STED and AFM provided further details. AFM resulted in less than 10% increase in mean fenestration size in comparison to STED. The effect might be ascribed to better AFM resolution – the PSF function causes blurring of the fenestration edges resulting in lower measured fenestration diameter. However, AFM imaging appeared to be prone to changes in mechanical properties. Data obtained using force tomography (a presentation of the image of the same area of LSEC reconstructed for increasing load force) showed load force dependence of fenestration size in living and FA/GA-fixed LSEC. The combined analysis of the elasticity and fenestration diameters for different loading forces indicate that increased cell rigidity aggravates tip-induced dilation of fenestrations. Cytochalasins reduce the Young’s modulus of LSEC [43] and the effect can be translated into the fixed cells too [56]. Cytochalasin B is known to promote actin depolymerization but without affecting actin cross-linking proteins [57]. These findings suggest that the change in LSEC elasticity after cytochalasin B treatment is mainly due to the reduction of actin stress fibers rather than disruption of actin mesh. Fenestra-associated cytoskeleton rings (FACR) within actin mesh are preserved after cytochalasin B treatment and the changes of fenestration size within FACR depend on the (not fully known up to date) structure building membrane and actin [1]. These data is in agreement with most LSEC studies with cytochalasin B reporting no changes in the fenestration diameter [13]. Therefore, we assume no influence of cytochalasin B on fenestration diameter, but the decreased elastic modulus and lack of stress fibers should be taken into consideration and imaging with cantilevers of low spring constant and use of load force close to the contact point should be applied to minimize the tip-induced deformation.

In general, all presented methods have their advantages and limitations, summarized in Table 1. We recommend the selection of microscopy techniques depending on the planned experiment. SIM and SEM provide higher throughput measurements of single cells or even groups of LSEC. SEM provides unprecedented resolution in the whole range of image size from individual sieve plates up to several LSEC. However, it requires sample drying and a relatively long sample preparation procedure. Therefore, SIM can be a first-choice method to quickly assess the effect of various drugs or toxins of LSEC. The limited resolution and lack of the ability to resolve fenestration in the perinuclear areas should not prevent the observation of relatively large changes in the porosity when cells are flat and cultured at low confluence. Its limitation is the resolution and (together with STED) the ability to resolve and measure fenestrations in perinuclear areas. STED allows a balance between the throughput (sample preparation plus measurement time) and the accuracy of measurements. Moreover, the optical techniques allow for simultaneous colocalization with other structures that can be additionally labeled. Both STED and AFM are scanning techniques which make them relatively slow. Large images of the whole LSEC are possible to obtain with some limitations. AFM requires up to 40 min per high-resolution image of a single LSEC while during long STED measurements (up to 8 min per LSEC), bleaching and thermal drift might hamper the imaging. Moreover, performing additional high-magnification images using AFM is required to obtain a resolution comparable with SEM. Both STED and AFM should be the best choice when small images of part of LSEC are analyzed for size measurements. Moreover, both were reported to be applicable for live LSEC imaging.

**Table 1:** Summary of presented microscopies.

	Wet fixed	Fixed dried	Fenestration size enlargement	Labeling	Live LSEC tracking	Applicability	Throughput	Measured feature size*	Accuracy
SEM	No	Yes	>30%	None	No	Whole cells and individual sieve plates	High	<10 nm	Number of fenestrations
							0.5–3 min/cell		
SIM	Yes^#^	–	None	Many dyes	Not reported yet	Whole cells and individual sieve plates	High	∼80 nm	Size of fenestrations
							0.5–3 min/cell		
STED	Yes^#^	–	None	Limited dyes	Yes	Whole cells	Moderate high	∼50 nm	Size of fenestrations
							5–8 min/cell		
						Individual sieve plates	High	∼50 nm	Size of fenestrations
							10–30 s/area		
AFM	Yes^†^	Yes	+10%**	None	Yes	Whole cells	Low	∼80 nm	Number of fenestrations
							25–40 min/cell		
						Individual sieve plates	Low	∼30 nm	Number of fenestrations
							2–5 min/area		Size of fenestrations

^*^The theoretical resolution limit of SIM on our system is ∼110 nm, but the combination with the thresholding of the image analysis yields a measurement size of ∼80 nm;

^**^For cantilevers with sharpened tip; dependent on the tip apex size;

^#^FA fixation recommended;

^†^GA fixation recommended.

SEM and AFM do not require sample labeling; hence the results provided by those techniques do not depend on the quality and density of labeling, making the analysis more reproducible. SIM can be used with almost all available dyes, while STED requires more specialized dyes. AFM can be used without any or with minimal sample preparation. We used this to our advantage to study the influence of each sample preparation step (required for other types of microscopies) on the topography of LSEC. Moreover, AFM remains the only technique showing fenestrations that are closed/fused within fenestrae-associated cytoskeleton rings (FACR) [58]. It also provides precise information about the height of investigated objects and allowing simultaneous assessment of the nanomechanical properties of investigated structures. Its main limitation is its low speed, which makes it impractical to use for large number of cells, as required, e.g., in screening of drug effects on LSEC morphology. On the other hand, AFM enables measurements of live LSEC and can be easily correlated with optical techniques. We therefore propose correlative live (AFM) and fixed (optical) imaging of LSEC. AFM lacks chemical information about investigated structures but provides unprecedented resolution for live LSEC imaging. The combination of AFM and fluorescence solves this problem. Here we propose that after the AFM experiment, when the dynamics of the structure of interest is captured, the sample can be fixed, labeled, and visualized using fluorescence microscopy. The resulting chemical information can be further extrapolated to the AFM data. We believe that such an approach in the near future would allow for a better understanding of the origin of fenestrations by disclosing the structure of fenestration-associated cytoskeletal structures as, e.g., fenestration-forming centers, defenestration centers, fenestration-associated cytoskeleton rings. Nowadays, the new dyes for live cell imaging are of great interest for researchers, but long-lasting fluorescence imaging options are limited. The main problems are related to phototoxicity, bleaching and dye-sample interaction [25]. The latter issue is significant for LSEC imaging as limited responsiveness of LSEC is a major issue in tracking cell membrane dynamics. The presented approach, alongside with AFM, can be used for high-resolution imaging of fenestrations in live LSEC to test drug responses.

## 5 Conclusions

We compared several techniques (SEM, SIM, STED, AFM) allowing for visualization of fenestrations in LSECs *in vitro* at the resolution allowing calculation of the fenestration diameter. We provided the first direct comparison of fenestrations in wet-fixed and fixed-dried LSEC. We showed that fenestration diameters measured using SEM are >30% larger than with other wet techniques. We advise using SEM to describe the number of fenestrations per cell. Fenestration diameters should ideally be calculated using wet-fixed samples. AFM provides nanometer range resolution in living and fixed LSECs ensuring useful coupling with the optical microscopy. Moreover, the construction of the AFM head makes it an easily applicable extension for most optical nanoscopy modalities that are based on inverted geometry. We provided the first comparison of FA/GA fixation on LSEC diameters measured with AFM. Glutaraldehyde fixation is highly advised for AFM to minimize the uncertainty of tip-induced sample alterations. Independent use of all presented techniques can be applied for measurements of fenestrations. However, their combination can provide novel, additional information from the correlative perspective, particularly AFM and conventional fluorescence microscopy, which combines high-resolution topographical information with cellular protein identification.

## Supplementary Material

Supplementary Material
