# Supplementary material for: From fixed-dried to wet-fixed to live – comparative super-resolution microscopy of liver sinusoidal endothelial cell fenestrations
Source: Nanophotonics. 2022 Apr 20;11(10):2253–70. doi: 10.1515/nanoph-2021-0818 (PMC11636152; doi:10.1515/nanoph-2021-0818)
Supplement: Supplementary file 1 — Supplementary Material [file j_nanoph-2021-0818_suppl.docx]

SUPPLEMENTARY INFORMATION

From fixed-dried to wet-fixed to live – comparative super-resolution microscopy of liver sinusoidal endothelial cell fenestrations

Karolina Szafranska^1^, Tanja Neuman^2^, Zbigniew Baster^3^, Zenon Rajfur^3^, Oskar Szelest^4^, Christopher Holte^1^, Agata Kubisiak^3^, Edyta Kus^5^, Deanna L. Wolfson^7^, Stefan Chlopicki^5,6^, Balpreet S. Ahluwalia^7^, Malgorzata Lekka^8^, Marek Szymonski^3^, Peter McCourt^1^, Bartlomiej Zapotoczny^1,8*^

^1^ Department of Medical Biology, Vascular Biology Research Group, University of Tromsø (UiT), The Arctic University of Norway, Tromsø, Norway

^2^ JPK BioAFM Business, Nano Surfaces and Metrology Division, Bruker Nano GmbH, Berlin, Germany

^3^ Marian Smoluchowski Institute of Physics, Faculty of Physics, Astronomy and Applied Computer Sciences, Jagiellonian University, Krakow, Poland

^4^ ICLab S.z.o.o, Krakow, Poland

^5^ Jagiellonian Centre for Experimental Therapeutics (JCET), Jagiellonian University, Krakow, Poland

^6^ Chair of Pharmacology, Jagiellonian University Medical College, Krakow, Poland

^7^ Department of Physics and Technology, UiT-The Arctic University of Norway, Tromsø, Norway

^8^ Institute of Nuclear Physics, Polish Academy of Sciences, Kraków, Poland

*Contact information

Corresponding author: Bartlomiej Zapotoczny. Tel.: +48 12 662 82 86.

e-mail address: bartlomiej.zapotoczny@ifj.edu.pl, (B. Zapotoczny).

Financial Support

This work is supported by the Polish National Science Centre under the “SYMFONIA 3” project, grant agreement no.: UMO-2015/16/W/NZ4/00070, the Research Council of Research Council of Norway Nano2021 program grant to “NanoChip”  Grant no. 288565, and the Polish National Science Centre under the “SONATA 15” Project, Grant Agreement No.: UMO-2019/35/D/NZ3/01804, and the European Union’s Horizon 2020 research and innovation program under the Marie Sklodowska-Curie grant agreement no. 766181, project “DeLIVER”

1. Cell isolation and sample handling

Firstly, livers were initially perfused to remove the blood and then digested using Liberase TM (*Roche)*. After digestion, the cells were released from Glisson’s capsule into a cold (4°C) perfusion buffer containing 1% BSA. The obtained suspension of cells was subjected to several centrifugations (including 25-50% Percoll gradient separation for AFM/STED experiments) to separate hepatocytes, remaining blood cells, and non-parenchymal cells. Thereafter, LSEC and Kupffer cells were separated by immuno-magnetic separation using endothelium specific CD146 MicroBeads (*MACS, Miltenyi Biotec*, Germany). After isolation, cells were seeded on uncoated glass coverslips and incubated in 5% CO_2_ at 37°C in EGM-2 cell culture medium (*Lonza*) for 12-15 hours for AFM/STED or on fibronectin-coated glass coverslips in RPMI-1640 (Sigma-Aldrich) for 4-6 hours for SIM/SEM, STED/SEM, and AFM/SEM. Seeding conditions were optimized according to the specific microscopy requirements, using established methodology [1]. Large gaps, i.e. micron-sized holes in a membrane, were observed in freshly isolated LSEC after seeding. By comparing live and fixed LSEC, we noticed that no new gap formation occurred when LSEC were fixed with 3.6% formaldehyde (FA) for 15 minutes or 1% GA for 2 minutes. By monitoring LSEC morphology live, instead of fixed, when using AFM/conventional fluorescence microscopy, we showed that wet-fixation of cultured LSEC does not damage sieve plates. However, by investigating individual LSEC using AFM on each step of sample preparation, we noticed that thorough rinsing (pipetting) of samples with live or fixed cells could damage the delicate structure of fenestrations within sieve plates. We used warm (37°C) buffers and fixative agents combined with slow aspiration and delicate rinsing to reduce gap formation.

2. More about the observation in correlative imaging of LSEC

In both SIM/SEM and STED/SEM we observed that visualization of thick areas of LSEC with optical methods is hampered by such “thickness”. Representative images are presented for SIM/SEM (**Supplementary figure 1**) and STED/SEM (**Supplementary figure 2**). The effect is a result of several factors. In contrast to SEM where imaging is done from the top, in both STED and SIM imaging is done in the inverted microscope setups with both excitation and emission beams coming from underneath the samples and cells. In STED, this forces the adjustment of the focal plane to the cell periphery area with fenestration (close to the substrate) and therefore fenestrations in the nuclear area are in the different focal plane, and thus not resolved properly. In SIM, the image reconstruction requires a series of z stacks at every 125 nm and then each plane is reconstructed. The images are not confocal so signal from the high/nuclear area where some fenestrations are observed are burdened with the signal from the rest of the cell body. For the final visualization we use projection images that average all z stacks which helps to improve signal to noise ratio and reduces background signal but it also disables observation of the objects that are not uniform throughout the whole cell. Another factor that causes this effect is the non-perpendicular orientation of those fenestrations, due to the slope from height difference between nucleus (2-3 μm) and cell periphery (0.3-0.5 μm).

Moreover, so called fenestration labyrinths are observed in these areas (**Supplementray figure 3**) which further interfere with imaging. As fenestrations are negative structures observed in optical techniques (i.e. we are looking for lack of a staining within fenestrations created by signal for the dye particles of the surrounding membrane), resolving fenestrations in these areas is overburdened with large errors.


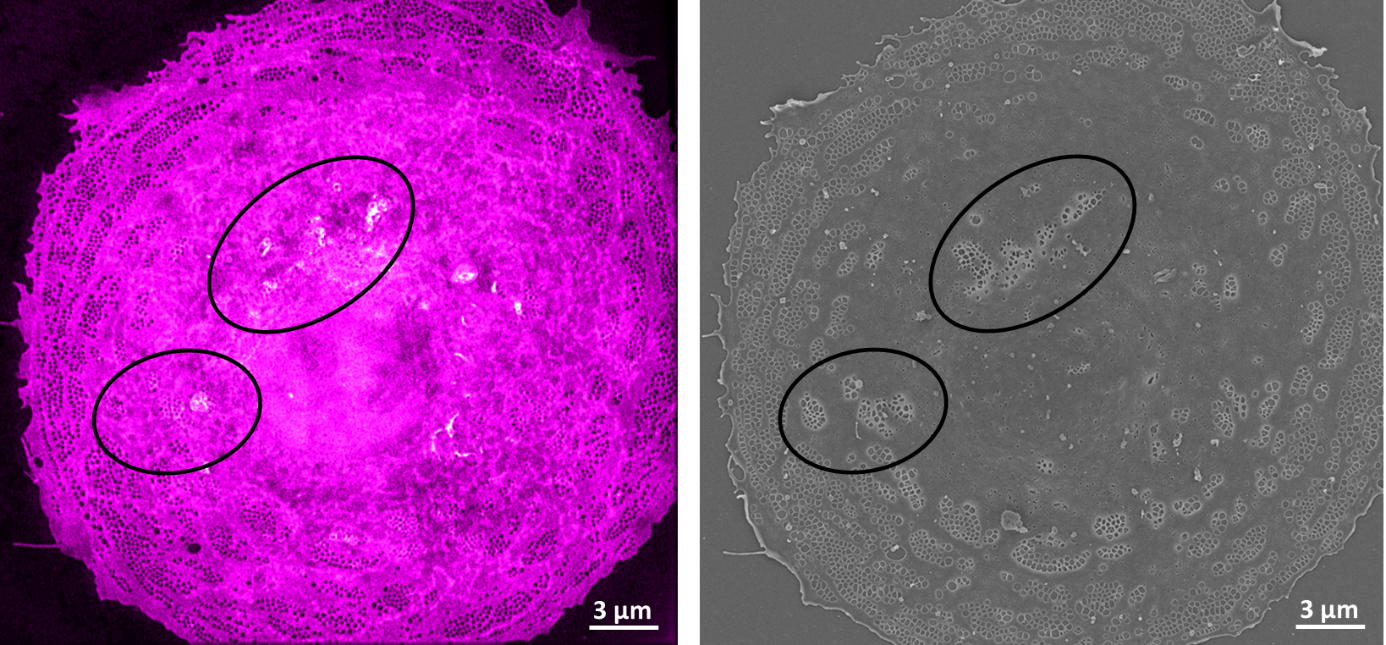


**Supplementary Figure 1** | Correlative SIM and SEM microscopy of a liver sinusoidal endothelial cell. Images present a representative LSEC analysed in Figure 1. The perinuclear zone, where the cell is thick, hampers visualization of fenestrations (encircled area). As a result, the number of fenestrations is underestimated in SIM.


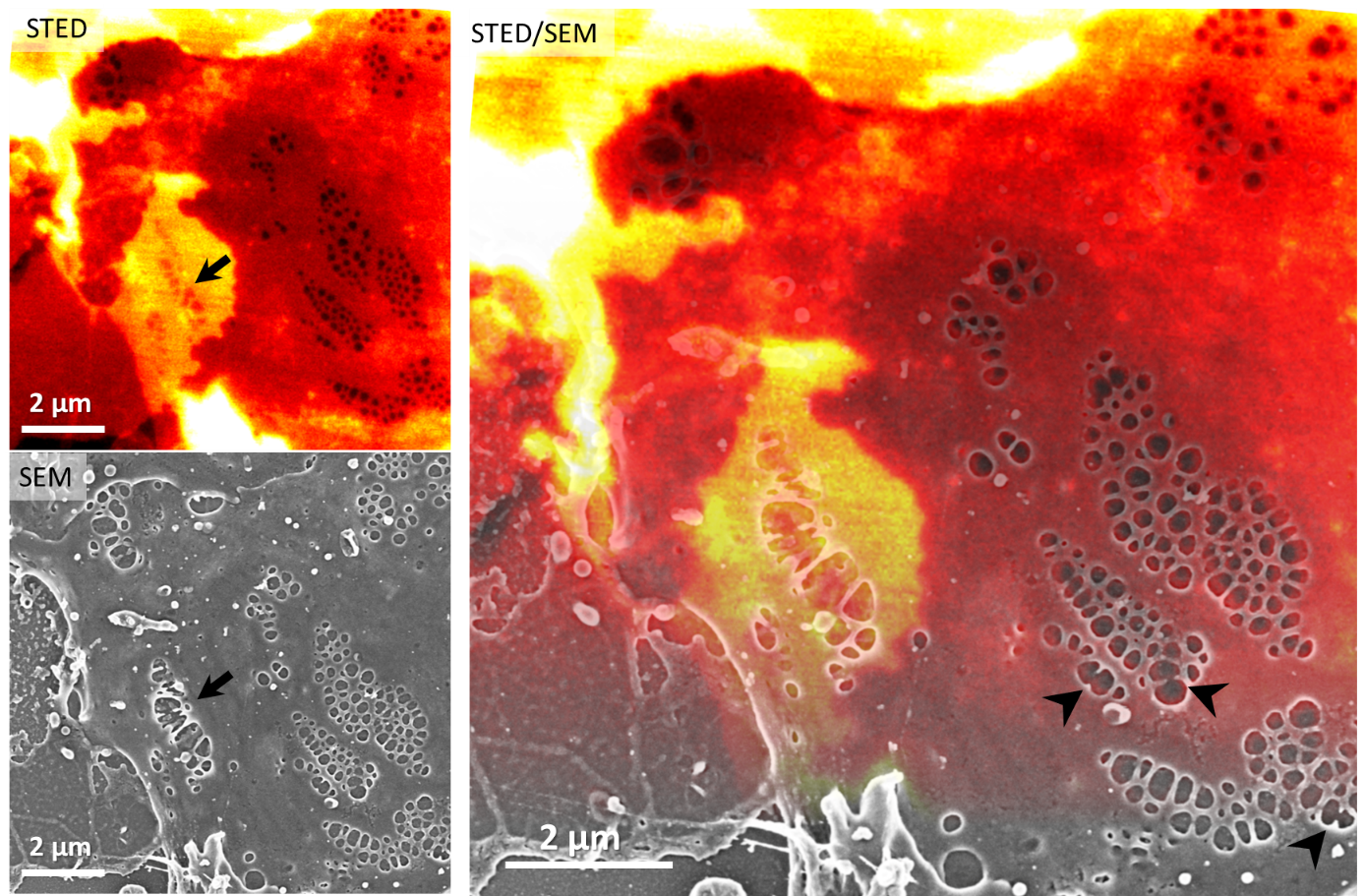


**Supplementary Figure 2** | Correlative STED and SEM microscopy of part of a LSEC. Images present a sieve plate that was selected for the analysis in Figure 2. The area of LSEC where the cell is thick hampers visualization of fenestrations (**arrow**). The same area is distorted in SEM, as dehydration affects these areas more than the flatter areas. The coalescence of fenestrations is also observed in SEM but not in STED (**arrowheads**).


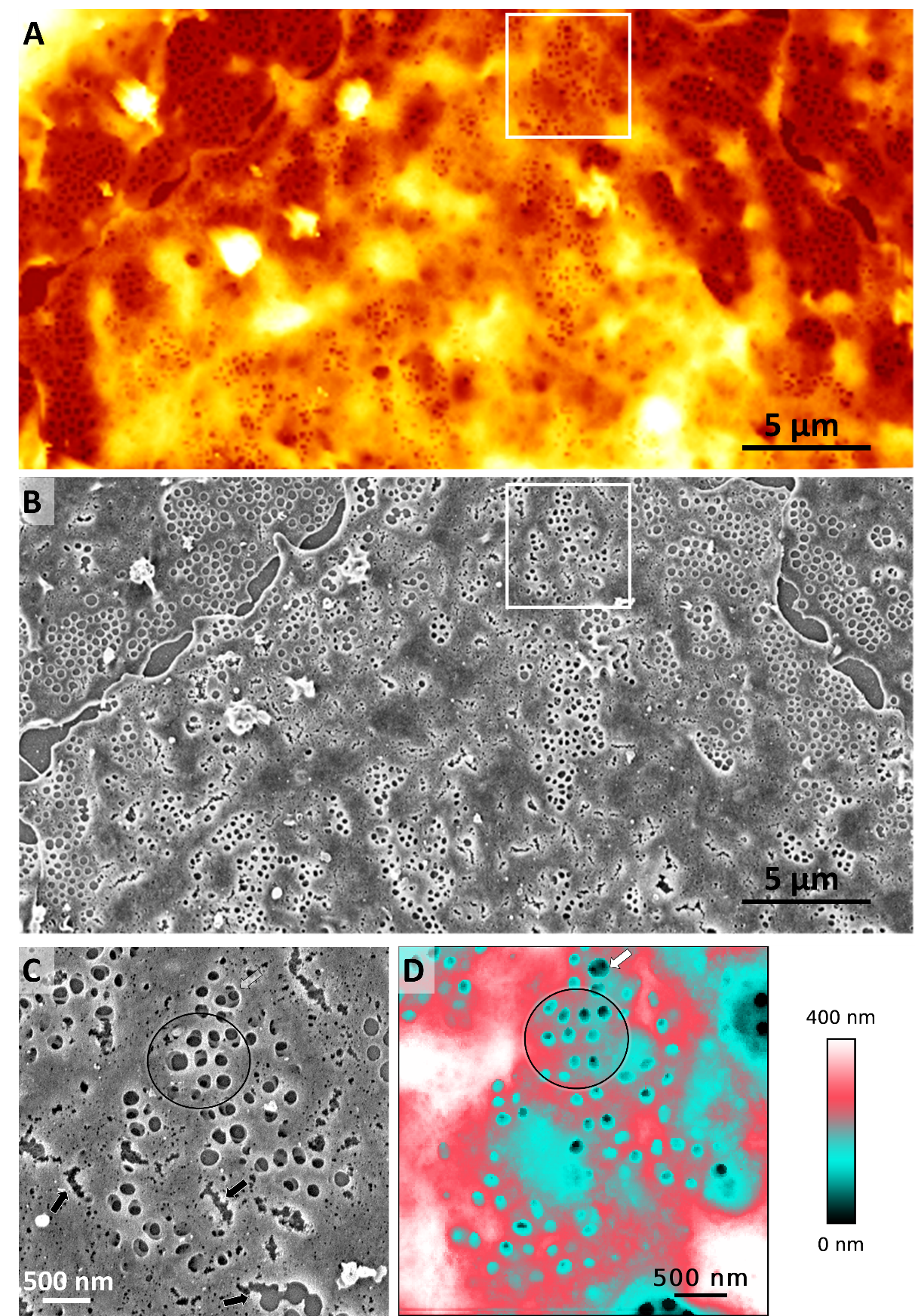


**Supplementary Figure 3** | Correlative AFM and SEM microscopy of parts of LSECs. Images present an interconnection of cells that was selected for the analysis in Figure 4. Large view AFM image (**A**) allows for identification of fenestrations and assessment of cell height and comparison with the corresponding area in SEM (**B**). The pixel-point (as introduced in[2]) of 80 nm does not allow for precise measurement of fenestration diameter. High-resolution AFM image analysis was performed in the selected area of the SEM micrograph. After dehydration cracks in cells are often observed (**black arrows**). (**C**). Encircled area in SEM (**C**) and AFM (**D**) highlight fenestration labyrinths – the areas in which fenestration lie over other fenestrations. Transcellular regions, where AFM tip reached glass substrate are presented as black. Rounded cyan-coloured areas indicate fenestrations, for which cell membrane was observed within them. In particular, within one top fenestration more than one fenestration can be observed in the bottom layer (**white arrow**).

2. The effect of permeabilisation on LSEC fenestrations

In order to investigate the influence of staining procedures on LSEC morphology, we stained LSEC with CellMask Deep Red and with phalloidin-Atto488, both preceded by 5 min 0.1% Triton X-100 treatment. We found the morphology of LSEC to be unaltered after staining; however, we observed significant changes in the morphology of fenestrations in permeabilized LSEC (**Figure 4**).


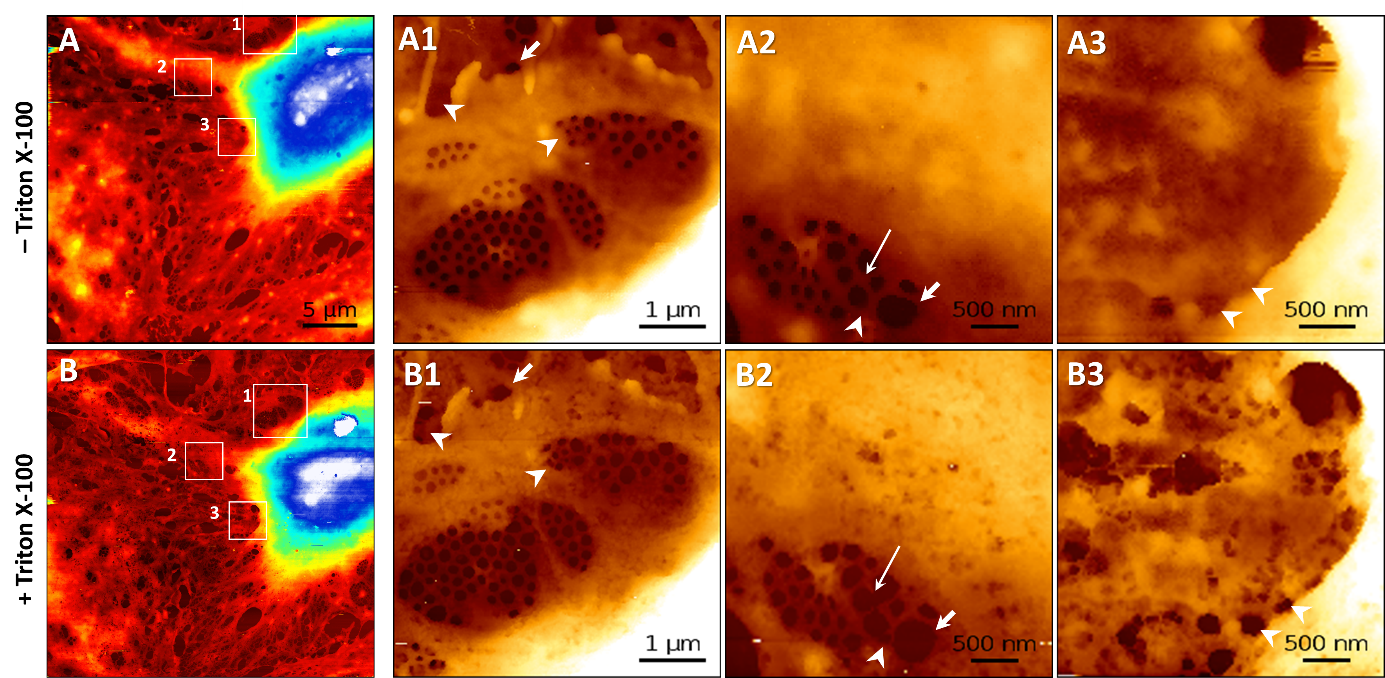


**Supplementary Figure 4** | Changes in the topography of LSEC in response to Triton X-100 treatment measured using QI AFM. **A,B** FA-fixed LSEC. **A1-3** Areas of interest were selected and measured in high resolution. **B** the same area as A, measured after treatment with 0.1% Triton X‑100 for 5 minutes. **B1-3** The same high-resolution images as in A1‑3 were selected after permeabilization. The formation of new fenestrations (**arrowhead**), enlargement (**short** **arrow**) and fusion (**long** **arrow**) of existing fenestrations was indicated. The same area was scanned twice to exclude the effect of AFM tip altering the samples – no changes induced by the AFM tip were observed.

Triton X-100 is a widely used detergent for permeabilization of the cell membrane [3]. It enables dyes and antibodies to reach the interior of the cell. Triton X-100 does not change the cortical cytoskeleton, and the overall cell morphology remains unchanged [4]. Nevertheless, even the slightest changes may influence the calculated dimensions when investigating objects at the nanoscale, such as fenestrations in LSEC. Indeed, we observed several new openings in the cell cytoplasm, and some of them were the size of fenestrations (**Figure 4, arrowhead**). Moreover, occasionally an enlargement in individual fenestrations was noticed, mainly due to coalescence of fenestrations (**Figure 4, arrows**). Similarly to another report [5], we did not observe any significant changes in cell elasticity after 0.1% Triton X-100 treatment.

The smooth cell membrane visualized with AFM for fixed cells became rough and distorted after 5 minutes of 0.1% Triton X-100 treatment. The merging of individual fenestrations and the formation of new open fenestrations were observed. However, the overall porosity seems to be only slightly affected, as most of the fenestrations gathered in sieve plates can be clearly identified. Nevertheless, we recommend using non-permeabilized cells when a detailed analysis of fenestration diameter is performed.

3. The effect of formaldehyde (FA) fixation on imaging of fenestrations in LSEC using AFM

Cytochalasin B was reported to not affect fenestration diameters. Here, we observed an increase in FA-fixed cells treated with cytochalasin B for the same load force used in the experiment (**Supplementary figure 5**). We observed higher load-force-dependence for cytochalasin B treated cells than for the control. Since the fenestration scaffold is made of actin [1],[6] and cytochalasin disrupts actin, the effect is expected for large load force values (here we used 350 pN). In **Figure 3** we showed that GA provides better crosslinking of proteins than FA resulting in higher apparent Young’s modulus values. When we analysed LSEC treated with cytochalasin B and fixed using GA the enlargement vanished. Therefore, we advise using GA-fixed LSEC (**Figure 4**), or use of minimal load force of < 100 pN (close to the contact point) (**Figure 5**), when fenestration diameters are analysed with AFM


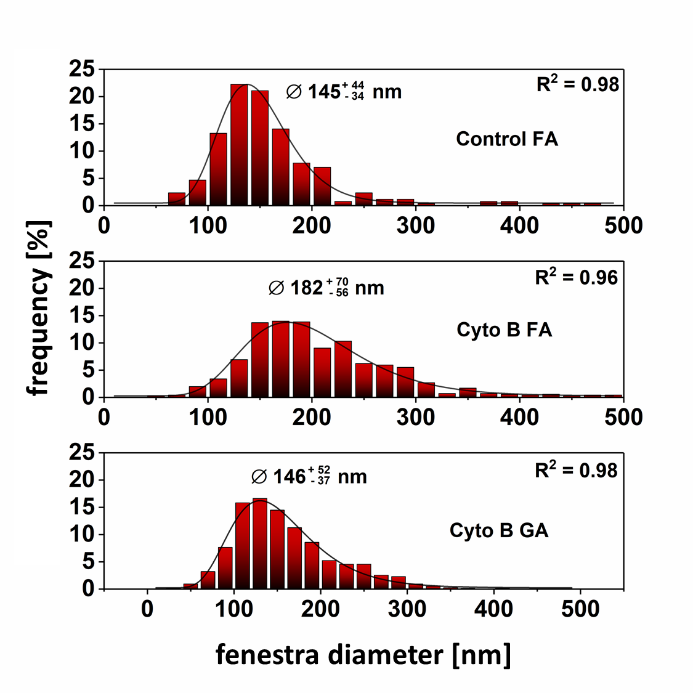


**Supplementary Figure 5** | The distribution of fenestration diameters obtained as a result of AFM measurements and analysis of LSEC fixed with glutaraldehyde (GA) and formaldehyde (FA), both control and treated with 21 µM cytochalasin B for 30 minutes prior to fixation. The load force of 350 pN was applied in all measurements (k=0.1, tip apex 25 nm). Mean values and standard deviation were presented above corresponding plot, resulting from log-normal fit.

Supplementary bibliography

[1] K. Szafranska et al., “Quantitative analysis methods for studying fenestrations in liver sinusoidal endothelial cells. A comparative study,” Micron, p. 103121, 2021, doi: https://doi.org/10.1016/j.micron.2021.103121.

[2] B. Zapotoczny, K. Szafranska, K. Owczarczyk, E. Kus, S. Chlopicki, and M. Szymonski, “Atomic Force Microscopy Reveals the Dynamic Morphology of Fenestrations in Live Liver Sinusoidal Endothelial Cells,” Sci. Rep., vol. 7, no. 1, p. 7994, 2017, doi: 10.1038/s41598-017-08555-0.

[3] M. Le Maire, P. Champeil, and J. V. Møller, “Interaction of membrane proteins and lipids with solubilizing detergents,” Biochim. Biophys. Acta - Biomembr., vol. 1508, no. 1–2, pp. 86–111, 2000, doi: 10.1016/S0304-4157(00)00010-1.

[4] X. Zhang, Q. Tang, L. Wu, J. Huang, and Y. Chen, “AFM visualization of cortical filaments/network under cell-bound membrane vesicles,” Biochim. Biophys. Acta - Biomembr., vol. 1848, no. 10, pp. 2225–2232, 2015, doi: 10.1016/j.bbamem.2015.06.025.

[5] K. B. Grimm, H. Oberleithner, and J. Fels, “Fixed endothelial cells exhibit physiologically relevant nanomechanics of the cortical actin web,” Nanotechnology, vol. 25, no. 21, 2014, doi: 10.1088/0957-4484/25/21/215101.

[6] V. Mönkemöller, C. Øie, W. Hübner, T. Huser, and P. McCourt, “Multimodal super-resolution optical microscopy visualizes the close connection between membrane and the cytoskeleton in liver sinusoidal endothelial cell fenestrations.,” Sci. Rep., vol. 5, p. 16279, 2015, doi: 10.1038/srep16279.
